# Reassessing Southern Ocean Air‐Sea CO_2_ Flux Estimates With the Addition of Biogeochemical Float Observations

**DOI:** 10.1029/2019GB006176

**Published:** 2019-11-16

**Authors:** Seth M. Bushinsky, Peter Landschützer, Christian Rödenbeck, Alison R. Gray, David Baker, Matthew R. Mazloff, Laure Resplandy, Kenneth S. Johnson, Jorge L. Sarmiento

**Affiliations:** ^1^ Program in Atmospheric and Oceanic Sciences Princeton University Princeton NJ USA; ^2^ Now at Department of Oceanography University of Hawai'i at Mānoa Honolulu HI USA; ^3^ Max Planck Institute for Meteorology Hamburg Germany; ^4^ Max Planck Institute for Biogeochemistry Jena Germany; ^5^ School of Oceanography University of Washington Seattle WA USA; ^6^ Cooperative Institute for Research in the Atmosphere Colorado State University Fort Collins CO USA; ^7^ Scripps Institution of Oceanography University of California, San Diego La Jolla CA USA; ^8^ Department of Geosciences and Princeton Environmental Institute Princeton University Princeton NJ USA; ^9^ Monterey Bay Aquarium Research Institute Moss Landing CA USA

**Keywords:** Southern Ocean, biogeochemical profiling floats, SOCCOM, global carbon cycle

## Abstract

New estimates of *p*CO_2_ from profiling floats deployed by the Southern Ocean Carbon and Climate Observations and Modeling (SOCCOM) project have demonstrated the importance of wintertime outgassing south of the Polar Front, challenging the accepted magnitude of Southern Ocean carbon uptake (Gray et al., 2018, https://doi:10.1029/2018GL078013). Here, we put 3.5 years of SOCCOM observations into broader context with the global surface carbon dioxide database (Surface Ocean CO_2_ Atlas, SOCAT) by using the two interpolation methods currently used to assess the ocean models in the Global Carbon Budget (Le Quéré et al., 2018, https://doi:10.5194/essd-10-2141-2018) to create a ship‐only, a float‐weighted, and a combined estimate of Southern Ocean carbon fluxes (<35°S). In our ship‐only estimate, we calculate a mean uptake of −1.14 ± 0.19 Pg C/yr for 2015–2017, consistent with prior studies. The float‐weighted estimate yields a significantly lower Southern Ocean uptake of −0.35 ± 0.19 Pg C/yr. Subsampling of high‐resolution ocean biogeochemical process models indicates that some of the differences between float and ship‐only estimates of the Southern Ocean carbon flux can be explained by spatial and temporal sampling differences. The combined ship and float estimate minimizes the root‐mean‐square *p*CO_2_ difference between the mapped product and both data sets, giving a new Southern Ocean uptake of −0.75 ± 0.22 Pg C/yr, though with uncertainties that overlap the ship‐only estimate. An atmospheric inversion reveals that a shift of this magnitude in the contemporary Southern Ocean carbon flux must be compensated for by ocean or land sinks within the Southern Hemisphere.

## Key Points

- A combined ship and float carbon dioxide flux estimate for the Southern Ocean yields 0.4 Pg C/yr less uptake than a ship‐only estimate
- Model subsampling indicates that some of the differences between ship and float flux estimates may be due to sampling times and locations
- An atmospheric inversion using the new ocean fluxes indicates that any compensating flux must be found in land or ocean south of 5°S

## 1 Introduction

The ocean has absorbed approximately 25% of anthropogenic carbon emissions released during the industrial era (Le Quéré et al., 2018). Present spatial assessments estimate that of the 2.6 ± 0.5 Pg C/yr of anthropogenic carbon dioxide dissolved in the ocean each year, over 40% enters in the Southern Ocean (Devries, 2014). While global climate models can be used to determine ocean and land carbon uptake (Le Quéré et al., 2018), atmospheric inversion estimates provide an independent, observation‐based method of partitioning contemporary terrestrial and ocean carbon fluxes (Peylin et al., 2013; Resplandy et al., 2018). Since the global sum of all carbon fluxes is well constrained from the rise in the atmospheric CO_2_ content, our estimates of both the mean and variability of oceanic carbon uptake indirectly impact inversion‐based estimates of terrestrial carbon uptake (Resplandy et al., 2018). Data‐based estimates of the ocean carbon sink also act as an assessment of global ocean model flux fidelity and therefore our ability to predict the rate of carbon dioxide increase in the surface ocean (Gruber et al., 2019; Le Quéré et al., 2018).

Observation‐based global air‐sea carbon flux estimates are traditionally determined from measurements of the partial pressure of carbon dioxide (*p*CO_2_) from ships or moorings. These measurements are then interpolated in time and space to produce global estimates of the *p*CO_2_ difference from the atmosphere and calculations of air‐sea flux (e.g., Landschützer et al., 2014; Rödenbeck et al., 2015; Takahashi et al., 2009). Shipboard observations of *p*CO_2_ are highly accurate (±2 μatm CO_2_ fugacity, Bakker et al., 2016), but many regions of the ocean remain sparsely sampled in time and space. Existing measurement density is biased toward the Northern Hemisphere and toward summer months. Integrated air‐sea flux estimates can also be determined from changes in oceanic carbon content, though infrequent repeat hydrographic sampling leaves these estimates unable to resolve short‐term variations in the air‐sea flux (Gruber et al., 2009; Gruber et al., 2019).

The primary basis for the contemporary Southern Ocean carbon flux estimate south of 35°S of ~ −1 Pg C/yr (Table 1, negative values indicate fluxes into the ocean) are the surface ocean observation‐based estimates of the air‐sea flux (e.g., Landschützer et al., 2017; Rödenbeck et al., 2013). This contemporary flux reflects the anthropogenic uptake overlaid on a natural flux that is thought to be roughly balanced between outgassing within and south of the Antarctic Circumpolar Current (ACC) and uptake to the north (Gruber, Landschützer, & Lovenduski, 2019). Westerly winds circling the Southern Ocean drive significant upwelling of old water enriched in dissolved inorganic carbon from centuries of organic matter remineralization. This carbon‐rich water drives an outgassing of natural carbon as the upwelled waters exchange with the atmosphere prior to sinking as either Antarctic Intermediate or Bottom Water (Gruber, Landschützer, & Lovenduski, 2019; Mikaloff Fletcher et al., 2007). North of the ACC, an uptake of natural carbon occurs, as surface waters are cooled prior to subduction as mode and intermediate waters. The outgassing south of approximately 55°S and the uptake between ~55°S and ~35°S are of approximately the same magnitude (0.5 Pg C/yr) but of opposite sign, roughly canceling each other and resulting in no net natural carbon flux (Gruber et al., 2009; Gruber, Landschützer, & Lovenduski, 2019; Mikaloff Fletcher et al., 2007). As a result, the contemporary flux is largely due to uptake of anthropogenic carbon.

**Table 1:** Southern Ocean Air‐Sea Carbon Flux From the Combined *p*CO_2_ Data Set Compared to Previous Estimates

Pg C/yr	Time period			
	1990–2000s		2015–2017	
	<44°S	<35°S	<44°S	<35°S
Ocean inversions, atmospheric inversion, or model estimates				
Gruber et al., 2009	−0.34 ± 0.2			
Lenton et al., 2013, a	−0.42 ± 0.07			

Mapped surface observations				
Lenton et al., 2013, b	−0.27 ± 0.13			
Landschützer et al., 2016, c	−0.24 ± 0.39	−0.79 ± 0.45	−0.41 ± 0.15	−1.1 ± 0.15
Rödenbeck et al., 2013, d	−0.44 ± 0.4	−0.95 ± 0.44	−0.63 ± 0.17	−1.19 ± 0.21
This study, SOCAT+SOCCOM^e^			−0.16 ± 0.18	−0.75 ± 0.22

Unmapped surface observations				
Gray et al. 2018^f^				−0.08 ± 0.55

a Lenton et al. (2013) estimate based on a mean of 26 models and inversions.

b Lenton et al. (2013) estimate based on surface *p*CO_2_ observations.

c Values calculated from the update through 2017 (Landschützer et al., 2017).

d Values calculated from the oc_v1.6 update through 2017 (10.17871/CarboScope‐oc_v1.6).

e The SOCCOM+SOCAT run from this study representing the mean ± 1 s.d. of the combined neural network and Jena CarboScope methods for 2015–2017. SOCCOM observations are used in this neural network run from April 2014 to December 2017, while SOCAT observations are from 1982–2017. This uncertainty overlaps with the SOCAT‐only uncertainty bounds.

f Gray et al. (2018) estimate is based on float *p*CO_2_ estimates from May 2014 to April 2017 with no interpolation.

The carbon flux in the Southern Ocean is subject to significant decadal variability. During the 1990s, Southern Ocean uptake is thought to have slowed relative to atmospheric emissions, before strengthening again in the 2000s to its current magnitude (Keppler & Landschützer, 2019; Landschützer et al., 2016; Le Quéré et al., 2007; Ritter et al., 2017). The initial weakening of Southern Ocean uptake may have been driven by changes in the strength and mean latitude of the westerlies and subsequent impacts on the amount of dissolved inorganic carbon‐rich waters upwelled in the Southern Ocean. The subsequent strengthening appears due to a combination of zonally heterogenous cooling and nonthermal changes in dissolved inorganic carbon and alkalinity (Gruber, Landschützer, & Lovenduski, 2019; Landschützer et al., 2015).

**Figure 1:**
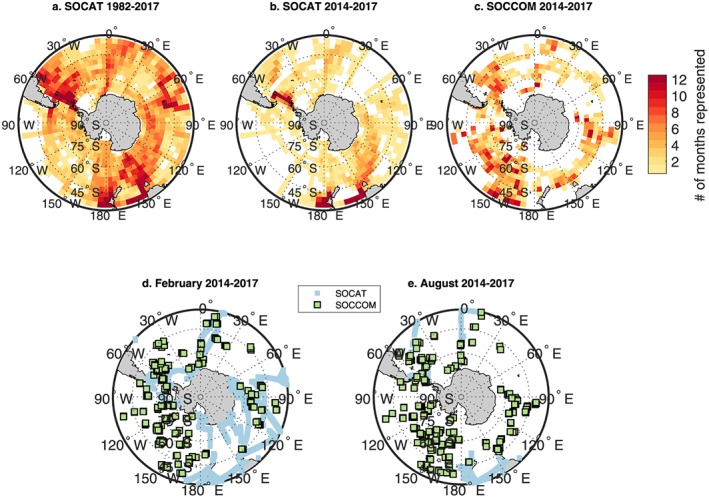
Shipboard and SOCCOM float sampling distributions. Temporal and spatial sampling density for (a) SOCAT: 1982–2017, (b) SOCAT: 2014–2017, and (c) SOCCOM. Colors represent the number of calendar months (maximum 12) with any data present in 3° latitude × 4° longitude grid cells. Grid cell size was chosen to approximate the decorrelation length scales of surface *p*CO_2_ in the Southern Ocean (Jones et al., 2012). The 1982–2017 SOCAT period represents the entire range of data used as inputs to the interpolation methods used in this study. Example summer (February, d) and winter (August, e) months for 2014–2017 illustrate the bias in shipboard observations toward warmer months as well as remaining gaps in SOCCOM float coverage. SOCCOM = Southern Ocean Carbon and Climate Observations and Modeling; SOCAT = Surface Ocean CO_2_ Atlas v6.

The Southern Ocean has few *p*CO_2_ observations, particularly in the wintertime (Figure 1). In 2014, the Southern Ocean Carbon and Climate Observations and Modeling (SOCCOM) project began deploying biogeochemical profiling floats aiming to fill this gap (Johnson et al., 2017), with 114 floats active as of December 2018. Using measurements of pH combined with a multiple linear regression‐derived alkalinity estimate, *p*CO_2_ can be calculated for each profile (uncertainty of ±2.86%, or 11.4 μatm at 400 μatm, Williams et al., 2017; Gray et al., 2018). Gray et al. (2018) used these new estimates of *p*CO_2_ to reveal a large outgassing of 0.36 Pg C/yr between the Polar Front and the maximum seasonal ice extent, where prior estimates calculated no net annual flux.

In this study, we estimate the Southern Ocean CO_2_ flux by combining the highly accurate shipboard measurements of *p*CO_2_ with observations from the expanding array of floats. We examine the impact of adding float data to the surface CO_2_ data sets and use high‐resolution model output to understand the influence of spatial and temporal sampling differences between ship and float‐based estimates. Using mooring observations, we present evidence that float observations made every 10 days are sufficient to resolve the annual air‐sea flux of carbon. Given that anthropogenic emissions are well known and the buildup of carbon in the atmosphere is well measured, a change in our estimates of the Southern Ocean contemporary carbon flux must be balanced by an equal and opposite change elsewhere. We therefore use an atmospheric inversion to determine which ocean or land regions are most likely to require a shift in flux to accommodate this novel Southern Ocean contemporary carbon flux estimate.

## 2 Methods

### 2.1 Regional Definitions

In this study, we divide the Southern Ocean into the Subtropical Zone (STZ), Subantarctic Zone (SAZ), Polar Frontal Zone (PFZ), Antarctic‐Southern Zone (ASZ), and Seasonal Ice Zone (SIZ) as defined in Gray et al. (2018). Briefly, the STZ is bounded to the north by 35°S and by the Subtropical Front (STF) to the south, defined by where the potential temperature (θ) at 100 m equals 11 °C. Moving south, the SAZ is the region between the STF and the Subantarctic Front (SAF), where θ at 400 m equals 5 °C. The PFZ is the region between the SAF and the Polar Front (PF), which is defined where θ equals 2 °C at the θ‐minimum between 0‐ and 200‐m depth. The ASZ is bounded by the PF to the north and the seasonal ice extent based on the mean 2014–2016 September 15% sea ice concentration location. The SIZ is the seasonally open water south of the 2014–2016 sea ice maximum. The STF, SAF, and PF were found by applying the above criteria to the Roemmich‐Gilson Argo‐based temperature and salinity climatology for 2014–2016 (Roemmich & Gilson, 2009). Sea ice extent was calculated from National Snow and Ice Data Center daily sea ice concentrations (NSIDC‐0051; Cavalieri et al., 1996). In this analysis the spatial extent of regions remains fixed through time. Overall Southern Ocean air‐sea fluxes presented in Table 1 are calculated for both <35°S, the northern boundary used in this study, and for <44°S, for ease of comparison with earlier work.

### 2.2 Surface‐Ocean Carbon Dioxide Data

Two sea surface carbon dioxide data sets were used in this study: (1) *p*CO_2_ measurements from underway shipboard and mooring data contained in the Surface Ocean CO_2_ Atlas v6 (SOCAT; Bakker et al., 2016; SOCAT data from 1982 to December 2017 used in this study) and (2) estimates of *p*CO_2_ from the SOCCOM biogeochemical float array (SOCCOM data through 31 December 2017, downloaded on 28 August 2018, Table S1 in the supporting information).

SOCCOM biogeochemical floats profile on a 10‐day cycle, measuring temperature, salinity, oxygen, pH, nitrate, fluorescence, and backscatter (Johnson et al., 2017). In situ float pH is adjusted at 1,500 m to ensure that observed pH is within 0.005 of the pH predicted from a multiple linear regression against temperature, salinity, pressure, nitrate, and oxygen (Williams et al., 2016). Alkalinity is estimated from temperature, salinity, nitrate, and oxygen using a multiple linear regression (Carter et al., 2018), and *p*CO_2_ is calculated as a function of pH and alkalinity. The estimated *p*CO_2_ has a calculated uncertainty of ±2.86% (11.4 μatm at 400 μatm) that is primarily due to an estimated uncertainty of 0.01 in the corrected pH (Gray et al., 2018; Williams et al., 2017).

**Figure 2:**
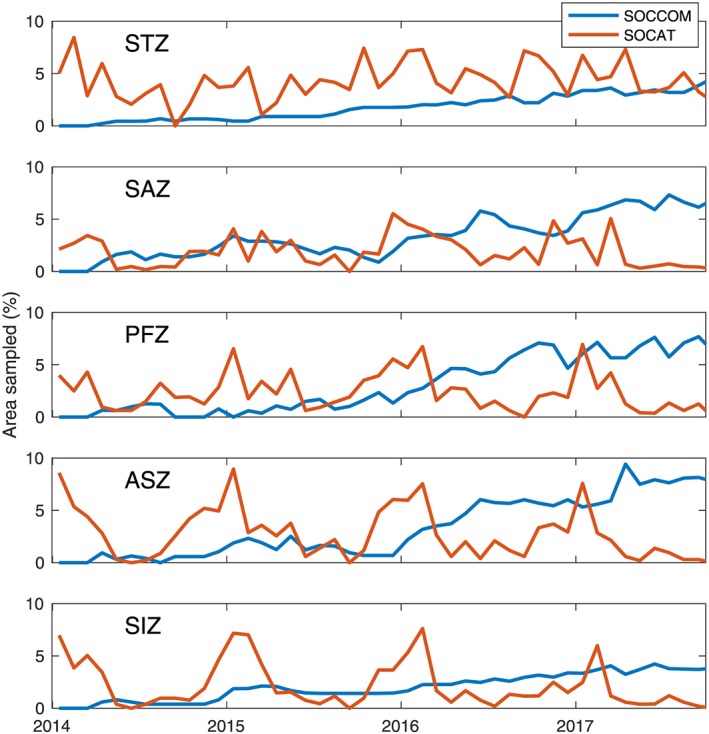
Seasonal and spatial sampling density of SOCCOM and SOCAT observations in the Southern Ocean. Area sampled per month is determined by counting the percent of 3° latitude × 4° longitude boxes with at least one observation in a given month. SOCAT observations (red lines) in the more southern regions are biased toward summertime sampling (year labels are on 1 January) and only seasonally does their coverage exceed 5% of a region's area in a given month. SOCCOM (blue line) float deployments began in April of 2014 and have rapidly increased sampling density in all seasons, surpassing area sampled by SOCAT in all regions except the STZ. SOCCOM = Southern Ocean Carbon and Climate Observations and Modeling; SOCAT = Surface Ocean CO_2_ Atlas v6; STZ = Subtropical Zone; SAZ = Subantarctic Zone; PFZ = Polar Frontal Zone; ASZ = Antarctic‐Southern Zone; SIZ = Seasonal Ice Zone.

Sampling density of independent measurements on time and space scales relevant to carbon observations were calculated by counting the number of 14‐day periods per month observed within each 3° latitude × 4° longitude grid cell (Figure 2). This grid size was chosen based on an approximate average autocorrelation length scale of 300 km for surface *p*CO_2_ in the Southern Ocean (Jones et al., 2012, Southern Ocean length scales range from ~30–1,200 km) in order to understand when additional sample locations are adding new information. There are millions more SOCAT observations than float samples. However, shipboard observations sample at high frequency, yielding samples close in both space and time that do not represent fully independent information for the purposes of understanding large‐scale fluxes. Choice of grid size impacts the calculated area sampled in this figure, as smaller boxes will give SOCAT observations relatively more area sampled due to the high frequency of measurements during ship transects.

### 2.3 Float Air‐Sea Carbon Dioxide Flux Calculations

To calculate air‐sea CO_2_ flux directly from float measurements, estimates of *p*CO_2_, latitude, longitude, temperature, and salinity from each profile were linearly interpolated to a 6‐hr time step. High‐resolution time steps are necessary to reproduce the nonlinear interaction between gas exchange and variability in wind speed (Wanninkhof, 2014). Six‐hourly wind speed and atmospheric pressure from the European Centre for Medium‐Range Weather Forecasts ERA‐Interim product (Dee et al., 2011) were spatially interpolated to the float locations. The air‐sea flux of CO_2_ was then calculated using the dry partial pressure atmospheric CO_2_ fraction from the Cape Grim Baseline Air Pollution Station (https://www.csiro.au/en/Research/OandA/Areas/Assessing-our-climate/Latest-greenhouse-gas-data) and the Wanninkhof (2014) gas flux parameterization. Float profiles were separated into each zone based on the geographic boundaries described in section 2.1. Monthly means for each region were calculated from available float data and annual means were then calculated from the resulting monthly data set. Uncertainties for the float‐derived air‐sea CO_2_ flux estimates were calculated from a 2,000‐iteration Monte Carlo analysis that contained ±20% uncertainty in the air‐sea gas flux parameterization (including uncertainty in wind speed, Wanninkhof, 2014) and a ±1.8% systematic bias and ±2.2% random uncertainty in the float‐derived *p*CO_2_ estimates (Gray et al., 2018).

### 2.4 Mapped Flux Analyses

This work focuses on two observation‐based interpolation methods for estimating global air‐sea carbon dioxide fluxes: a neural network approach that determines the relationships between *p*CO_2_ and environmental variables (Landschützer et al., 2013) and a biogeochemical interpolation scheme tuned to fit surface *p*CO_2_ estimates (Rödenbeck et al., 2013). Both methods were run using three data set versions: one based on SOCATv6 alone (“SOCAT‐only”), one using observations from both SOCAT and SOCCOM (“SOCAT+SOCCOM”), and a “SOCCOM‐weighted” data set using both data sets but excluding all SOCAT data south of 35°S in 2014 and thereafter. The SOCCOM‐weighted data set was tested in order to better understand the differences between the SOCAT data set and the new SOCCOM‐based *p*CO_2_ estimates. The number of SOCCOM observations is increasing and, as this is the first approach combining these two data sets, it is important to test the impact of weighting SOCCOM observations more heavily as a possible end point to their influence. A test of sensitivity to possible bias in the SOCCOM *p*CO_2_ estimates was performed by repeating the SOCAT+SOCCOM and SOCCOM‐weighted runs with the SOCCOM *p*CO_2_ estimates artificially lowered by 4 μatm, based on the mean offsets found in cross‐over analyses of nearby float and ship measurements (Fay et al., 2018; Gray et al., 2018; Williams et al., 2018).

The Landschützer et al. (2013) approach builds on a two‐step neural network technique (self‐organizing map feed‐forward network, SOM‐FFN) to extrapolate the available *p*CO_2_ measurements from the gridded SOCATv6 data set (Bakker et al., 2016; Sabine et al., 2013). Available float measurements falling within 1° × 1° grid cells in a given month were averaged for each data set. In the combined SOCAT+SOCCOM run, for any month where SOCAT and SOCCOM observations existed in the same 1° × 1° grid cell, the two monthly values were averaged together, giving SOCAT and SOCCOM observations equal weight in that one grid cell. Based on statistical relationships with independent proxy variables from satellite and reanalysis products (see Landschützer et al., 2018 for most recent details), the neural network provides global maps of the surface ocean *p*CO_2_. The monthly air‐sea CO_2_ flux is then calculated from the difference between the surface ocean *p*CO_2_ and the atmospheric *p*CO_2_ calculated from the NOAA Marine Boundary Layer dry air mixing ratio of atmospheric CO_2_ (https://www.esrl.noaa.gov/gmd/ccgg/mbl/) and corrected for water vapor pressure (see Dickson et al., 2007). The gas transfer is calculated from a quadratic gas transfer velocity relationship scaled to a mean transfer velocity of 16 cm/hr(Wanninkhof, 2014) and ERA‐Interim reanalysis winds.

Complementary to the neural network mapping, the Jena CarboScope *p*CO_2_ mapping scheme does not involve any regression of *p*CO_2_ against driving variables but simply interpolates the *p*CO_2_ data in space and time according to prescribed spatial and temporal autocorrelations (Rödenbeck et al., 2013, 2014). In order to ensure that the interpolated *p*CO_2_ field is also compatible with the dynamics of mixed‐layer carbon content (including the buffer effect), this is done in the following steps: First, the sea‐air CO_2_ fluxes and the *p*CO_2_ field are linked numerically to the spatiotemporal field of ocean‐internal carbon sources/sinks through parametrizations of sea‐air gas exchange, solubility, and carbonate chemistry, as well as a budget equation for mixed‐layer dissolved inorganic carbon. Then, the ocean‐internal carbon sources/sinks are adjusted to optimally fit the *p*CO_2_ field to the *p*CO_2_ observations. The spatiotemporal interpolation is achieved by Bayesian a priori smoothness constraints with prescribed spatial and temporal decorrelation scales. Temporal interpolation also results from the inherent relaxation time scales of the mixed‐layer carbon budget. The calculation operates on daily time steps and 4° × 5° pixels, to which the *p*CO_2_ data are binned before use. For the SOCAT‐only run of this study, we use the CarboScope interpolation run based on SOCATv6 (CarboScope ID oc_v1.6), as well as analogous runs based on the combined SOCAT+SOCCOM data set (oc_SOCCOM_v1.6) or on the SOCCOM‐weighted data set (oc_SOCCOMonly_v1.6).

Both mapping methods were run for the period 1982–2017. To avoid the influence of earlier years with no float data, we primarily analyze monthly mean fluxes from 2015–2017, leaving out the 2014 fluxes, for which the float data were relatively sparse. This allows us to focus on the period when SOCCOM and SOCAT data best overlap and examine the influence of the float data on these mapping products.

Uncertainty estimates (±1 s.d.) for the mapped CO_2_ flux products represent a combined standard deviation of the interannual variability and a method uncertainty of ±0.15 Pg C/yr over the whole Southern Ocean for each mapping method.

### 2.5 Model Subsampling

To examine the impact of the different spatial and temporal coverage of the SOCCOM and SOCAT data sets, we tested the sampling differences using subsampled output from two models: (1) CM2.6 from the NOAA Geophysical Fluid Dynamics Laboratory (Delworth et al., 2012; Galbraith et al., 2015) and (2) the Southern Ocean State Estimate (SOSE, Mazloff & Verdy, 2015; Verdy & Mazloff, 2017). CM2.6 is an eddy‐resolving model with an ocean horizontal resolution of 1/10° with 50 vertical layers and the miniBLING embedded biogeochemical model. We use the last 20 years of an 80‐year run with a 1% atmospheric CO_2_ increase per year after a 120‐year spin‐up. The 1% increase per year experiment was chosen to simulate a scenario where water upwelled in the Southern Ocean had last been exposed to a lower atmospheric *p*CO_2_ than at the time of current ventilation. SOSE is a data‐assimilating state estimate with an ocean resolution of 1/6° and 52 vertical layers, physics based on the MITgcm, and the BLINGv2 biogeochemical model (Verdy & Mazloff, 2017). Iteration 106 of the 2008‐2012 solution and iteration 122 of the 2013–2017 solution were used from SOSE, where the iteration number identifies a specific solution as the model is optimized based on observations. SOSE assimilates SOCATv5 and Argo data, including biogeochemical parameters from the SOCCOM float array.

CM2.6 is a climate model and does not represent specific historical years, while output from SOSE represents 2008–2017. For both models, our goal was to use the modeled output as a realistic representation of the temporal and spatial patterns of air‐sea CO_2_ fluxes, to determine how the SOCAT and SOCCOM data sets might be biased due to their respective sampling distributions. To do this, we took the times and locations of SOCCOM and SOCAT observations from 1998–2017 (2008–2017 for SOSE) and subsampled daily *p*CO_2_ output from both models. The subsampled daily output was then mapped using the SOM‐FFN approach of Landschützer et al. (2016). Subsampled daily output was gridded into 1° × 1° monthly means for SOCAT‐only, SOCCOM+SOCAT, and SOCCOM‐weighted runs, similar to the real‐world approach described above. To create the biomes required in the first step of the SOM‐FFN method, monthly means of model sea surface temperature, sea surface salinity, a mixed‐layer depth (MLD) climatology, and a surface *p*CO_2_ climatology were input to the SOM. Sixteen biomes were used for the global CM2.6 output, while nine biomes were used for SOSE output which only spans 29.5°S to 90°S. Monthly mean fields of sea surface temperature, sea surface salinity, climatological MLD, chlorophyll, and atmospheric *p*CO_2_ were used to establish relationships with *p*CO_2_ pseudo‐observations and to subsequently map *p*CO_2_. Air‐sea fluxes were calculated from mapped *p*CO_2_ using the same approach as in section 2.4. For creation of the MLD and *p*CO_2_ climatologies, a mean seasonal cycle was calculated from the 10‐ or 20‐year model output.

One key difference between this modeling exercise and use of the SOM‐FFN with real observational data is that the inputs are perfect. This is especially important for the *p*CO_2_ climatology that is input to the SOM for biome creation. The Landschützer et al. (2016) approach utilizes the Takahashi et al. (2009) climatological map, which is based on in situ observations and mapped using an advection‐based interpolation scheme. Because this is likely the least well constrained input to the SOM step and adds significant information regarding which regions of the Southern Ocean should be expected to have similar relationships between *p*CO_2_ and predictor variables, we tested the SOM‐FFN approach for CM2.6 both with and without the *p*CO_2_ climatology as an input to the SOM step.

## 3 Results/Discussion

### 3.1 Carbon Dioxide Flux Estimates

The three carbon flux products produced using the Landschützer et al. (2013) neural network and the Rödenbeck et al. (2013) Jena CarboScope interpolation scheme illustrate the impact of adding floats to the SOCAT‐based estimates of carbon dioxide fluxes. Figures for the neural network are presented here; equivalent figures for the interpolation scheme are found in the supporting information.

**Figure 3:**
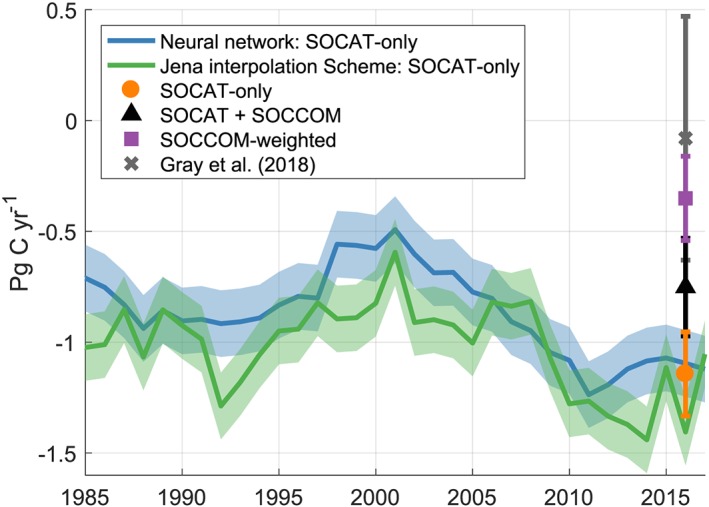
Neural network and Jena CarboScope‐derived Southern Ocean (south of 35°S) air‐sea carbon dioxide fluxes. Addition of SOCCOM floats to the mapping product‐derived CO_2_ fluxes reduces the mean 2015–2017 Southern Ocean carbon sink from −1.14 ± 0.19 Pg C/yr (orange circle) to −0.75 ± 0.22 Pg C/yr (black triangle), and removal of shipboard observations reduces the sink by an additional 0.4 Pg C/yr to −0.35 ± 0.19 Pg C/yr (purple square). Symbols and error bars represent the average of both products and the combined standard deviation of interannual variability, differences between the means of each product, and a method uncertainty of ±0.15 Pg C. The nonmapped, float‐only estimate of Gray et al. (2018) is included for reference (gray “x”). SOCCOM = Southern Ocean Carbon and Climate Observations and Modeling; SOCAT = Surface Ocean CO_2_ Atlas v6.

The annual mean Southern Ocean carbon flux from the SOCAT‐only *p*CO_2_ product varies between −1.1 and −1.25 Pg C/yr over the past 4 years (negative into the ocean, Figure 3 and Table 2). The SOCCOM‐weighted product diverges significantly, with an uptake of −0.87 ± 0.26 Pg C/yr in 2014 that decreases to −0.26 ± 0.16 Pg C/yr in 2017 (Table 2). The combined SOCAT+SOCCOM product is in between the individual products, with a flux into the ocean of −0.61 ± 0.26 Pg C/yr in 2017, or a reduction in uptake of 0.48 Pg C/yr from the SOCAT‐only product. The mean 2015–2017 fluxes over the entire Southern Ocean are −1.14 ± 0.19, −0.75 ± 0.22, and −0.35 ± 0.19 Pg C/yr for the SOCAT‐only, SOCAT+SOCCOM, and SOCCOM‐weighted cases, respectively. Note that at this early stage of the SOCCOM project, the uncertainty bars of these estimates overlap. The SOCCOM‐weighted product overlaps with the Gray et al. (2018) uptake of −0.08 ± 0.55 Pg C/yr for 1 May 2014 to 30 April 2017, in part due to the large uncertainty interval for the noninterpolated Gray et al. approach (Figure 3).

**Table 2:** Annual Southern Ocean Carbon Fluxes From Three *p*CO_2_ Products^a^

Southern Ocean	SOCAT	SOCAT + SOCCOM	SOCCOM
(Pg C/yr_,_ south of 35°S)			
2014	−1.26 ± 0.23	−1.17 ± 0.24	−0.87 ± 0.26
2015	−1.09 ± 0.15	−0.83 ± 0.16	−0.47 ± 0.17
2016	−1.25 ± 0.22	−0.82 ± 0.15	−0.32 ± 0.17
2017	−1.09 ± 0.15	−0.61 ± 0.26	−0.26 ± 0.16

a Fluxes presented here represent the average of the neural network and Jena CarboScope interpolation scheme mapping methods. Uncertainties represent ±1 s.d. and were calculated as the combined standard deviation of the mapping product differences and a ± 0.15 Pg C/yr uncertainty for each method.

**Figure 4:**
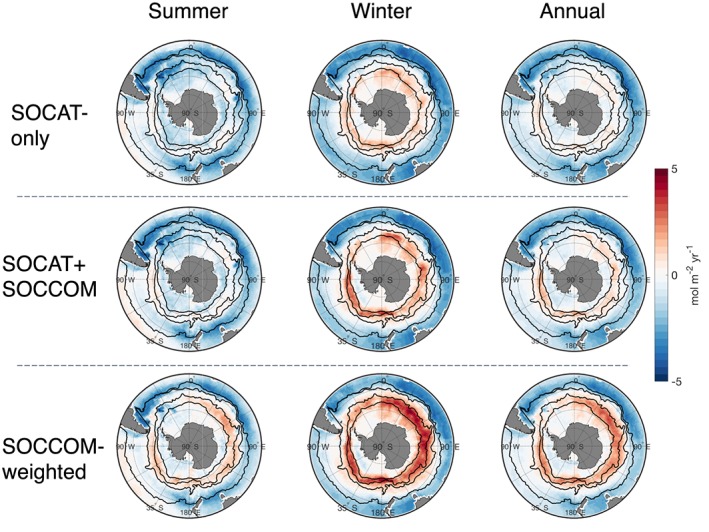
Mean 2015–2017 summer, winter, and annual Southern Ocean fluxes from all three *p*CO_2_ products. Fronts and boundaries shown as black lines are (from north to south) as follows: the subtropical front, subantarctic front, polar front, and seasonal ice extent. Neural network‐derived fluxes show strong summer (November to April) uptake in the SOCAT‐only product combines with moderate winter (May to October) outgassing around the Polar Front to give an annual flux with little outgassing evident. Addition of the SOCCOM floats produces stronger outgassing in the winter SOCAT+SOCCOM map, and has a moderate impact on the mean annual flux. The SOCCOM‐weighted product displays outgassing around the Polar Front even in the summer and a very strong winter signal. Equivalent fluxes for Jena interpolation scheme in Figure S1 in the supporting information. SOCCOM = Southern Ocean Carbon and Climate Observations and Modeling; SOCAT = Surface Ocean CO_2_ Atlas v6.

**Figure 5:**
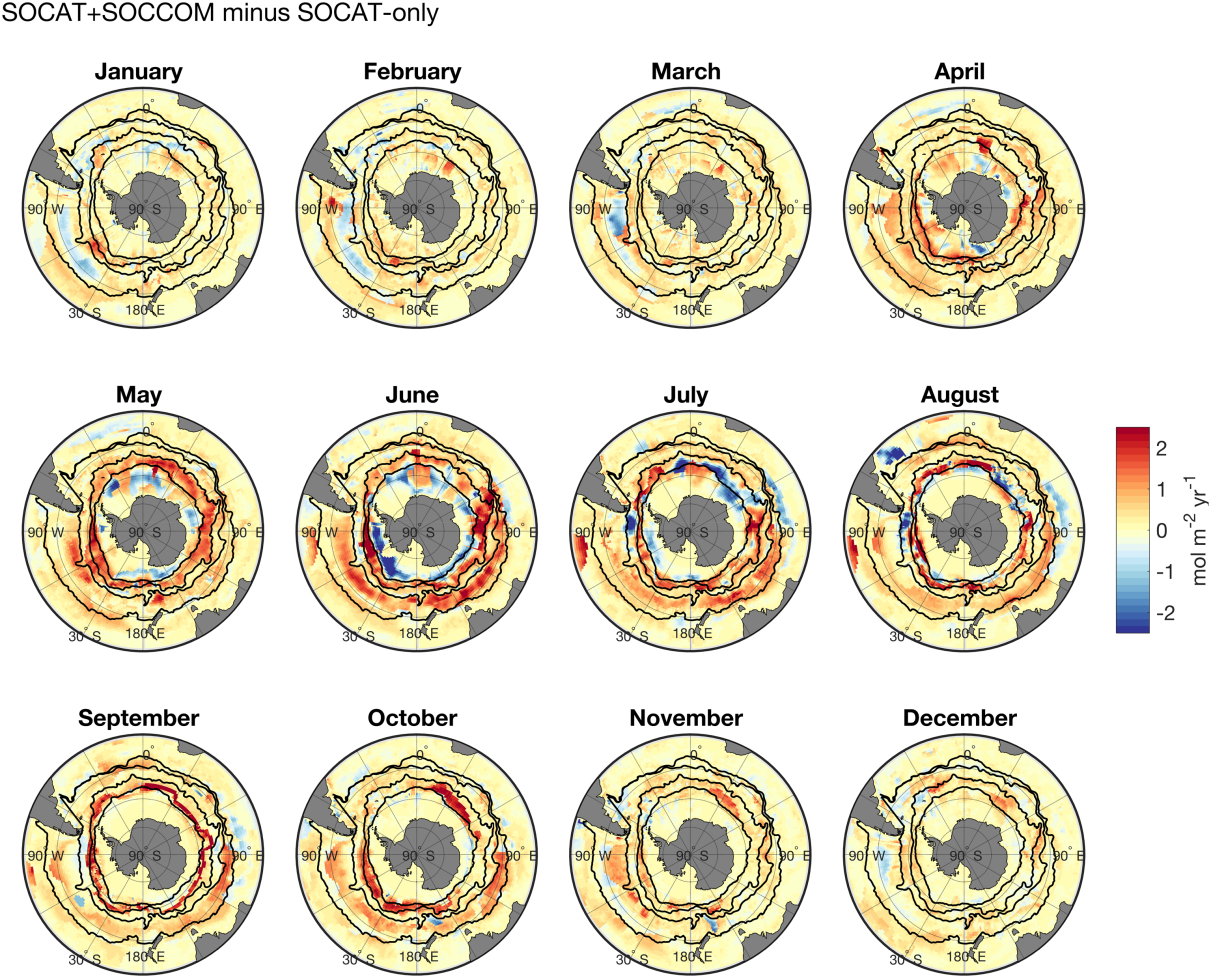
Impact of adding SOCCOM floats on neural network CO_2_ fluxes. SOCAT+SOCCOM monthly mean air‐sea CO_2_ fluxes (2015–2017) minus SOCAT‐only monthly mean fluxes. Red values indicate regions where addition of float‐estimated *p*CO_2_ decreased the Southern Ocean sink. Equivalent figure for the Jena interpolation scheme in supporting information (Figure S2). SOCCOM = Southern Ocean Carbon and Climate Observations and Modeling; SOCAT = Surface Ocean CO_2_ Atlas v6.

Summer, winter, and annual mean flux maps from the 2015–2017 results indicate that the addition of float observations enhances wintertime outgassing in the region around the ACC (Figures 4 and S1). The addition of floats in the SOCAT+SOCCOM product has little impact on summertime fluxes. A larger change in summertime fluxes is present in the SOCCOM‐weighted product, though still with the majority of the change in annual fluxes due to the increased wintertime outgassing. Mean monthly air‐sea flux difference maps further indicate where and when float observations are impacting the calculated air‐sea fluxes. Subtracting SOCAT‐only from SOCAT+SOCCOM (Figure 5) indicates that addition of the float data has the largest impact in the wintertime where the SOCAT data set is sparsest, that is, centered around the Polar Front in the Antarctic‐Southern and Polar Frontal Zones. In contrast, there is little impact during the summer months and in more northerly regions. Corresponding figures for the Jena CarboScope interpolation scheme indicate similar patterns, though with a stronger impact occurring over more of the year (Figure S2).

**Figure 6:**
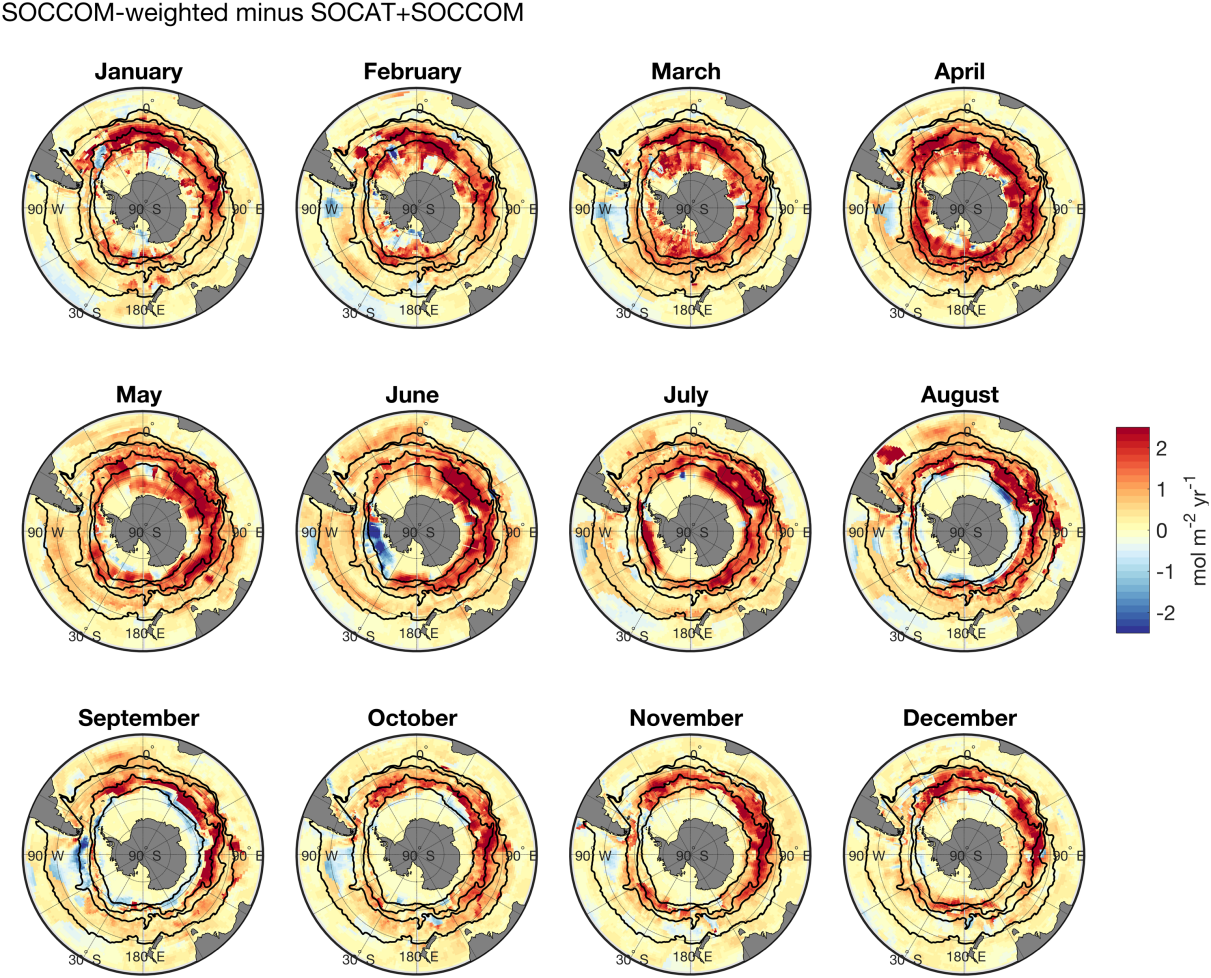
Impact of removing SOCAT shipboard data on neural network CO_2_ fluxes. SOCCOM‐weighted monthly mean air‐sea CO_2_ fluxes (2015–2017) were subtracted from SOCAT+SOCCOM monthly mean fluxes. Red values indicate regions where removal of ship‐measured *p*CO_2_ decreased the Southern Ocean sink or increased outgassing. Equivalent figure for the Jena interpolation scheme in supporting information (Figure S3). SOCCOM = Southern Ocean Carbon and Climate Observations and Modeling; SOCAT = Surface Ocean CO_2_ Atlas v6.

Float observations alone are, however, insufficient to recreate all the important features of the annual carbon dioxide cycle in the Southern Ocean. By subtracting the SOCCOM‐weighted product from the combined SOCAT+SOCCOM product (Figure 6), we find differences in the summertime over broad regions further north in the PFZ and SAZ. If float observations alone were currently adequate to constrain the relationships between the CO_2_ flux and the predictor variables in the Southern Ocean, removing the SOCAT data set would be expected to result in a negligible difference. The large change in calculated CO_2_ flux between the SOCCOM‐weighted and SOCAT+SOCCOM product indicates that the CO_2_ flux in the Southern Ocean is currently underconstrained by float data alone, or that the SOCCOM and SOCAT data sets are significantly different and that the combined data set is weighted more toward the shipboard observations. We address some possible differences between the two data sets in section 3.2. For the equivalent analysis as Figures 5 and 6, the differences in the Jena CarboScope results indicate greater zonal patchiness, likely due to the influence of SOCCOM data in areas with few SOCAT observations (Figures S2 and S3). Such zonal heterogeneity is more strongly seen in the CarboScope interpolation having explicit zonal resolution, while the biogeochemical provinces produced by the neural network link areas of similar sea surface properties, tending toward a more uniform zonal response.

**Figure 7:**
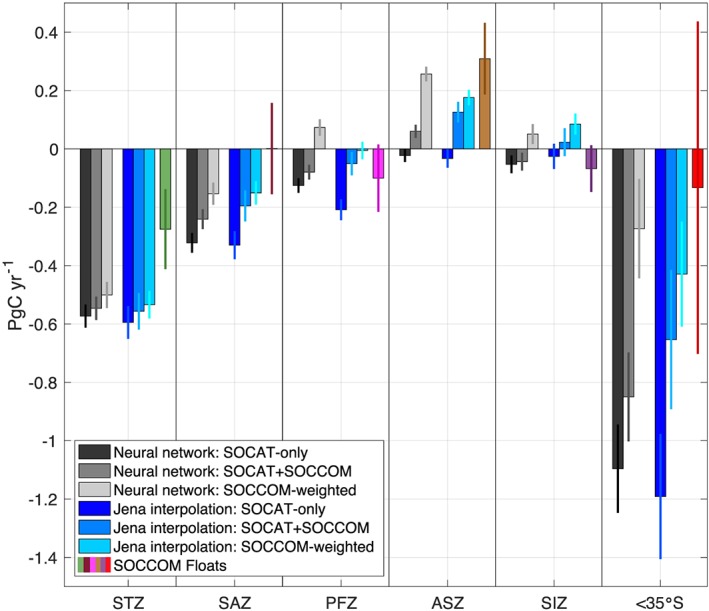
Mean annual fluxes for Southern Ocean regions from 2015–2017 for two data‐based products and a float‐only average. Neural network fluxes (grays), Jena mixed layer scheme (blues), and float‐only fluxes (colors) all indicate a reduction in the Southern Ocean carbon sink as the influence of the float observations is increased. The largest impacts are in the Subantarctic Zone (SAZ), Polar‐Frontal Zone (PFZ), and Antarctic‐Southern Zone (ASZ), which combine with smaller differences in the fluxes of the other regions to yield a large overall impact in the total Southern Ocean. Error bars represent ±1 s.d. SOCCOM = Southern Ocean Carbon and Climate Observations and Modeling; SOCAT = Surface Ocean CO_2_ Atlas v6.

Regional annual mean 2015–2017 fluxes from the neural network and interpolation scheme for all three *p*CO_2_ products and the float‐only averages are presented in Figure 7. The largest differences in the regional mean fluxes between the SOCAT‐only and SOCAT+SOCCOM neural network products are in the SAZ and ASZ, with roughly 0.1 Pg C/yr change in both. The Jena interpolation scheme produces a similar pattern, though with a greater magnitude. In the Jena estimates, addition of the float *p*CO_2_ estimates reduces carbon dioxide uptake in the SAZ, PFZ, and ASZ by 0.13, 0.16, and 0.16 Pg C/yr, respectively, yielding a more significant impact on the total Southern Ocean flux of −0.54 Pg C/yr. The removal of shipboard data causes a reduction in uptake in the ASZ of an additional 0.2 Pg C/yr in the neural network product, a slightly smaller reduction in the PFZ, and changes of ~0.1 Pg C/yr in the SAZ and SIZ. Overall, while the biggest total regional differences between the SOCAT‐only and SOCCOM‐weighted products occurs in the ASZ, in broad agreement with the analysis of Gray et al. (2018), we find significant changes in all regions other than the STZ, and all toward a reduction in the Southern Ocean carbon sink. It is important to recognize that the STZ and SAZ regions are most likely to be influenced by SOCAT observations outside of the Southern Ocean, and therefore may have a more muted response to the addition of SOCCOM observations.

Two possibilities arise for the reduction in Southern Ocean uptake with the addition of float observations: (1) the float‐derived estimates of *p*CO_2_ are biased high, resulting in a reduction of the air‐sea *p*CO_2_ gradient that drives gas into the ocean, and (2) the differences in sampling locations and times between the SOCAT and SOCCOM data sets result in different waters sampled. Comparisons between *p*CO_2_ from underway ship measurements and float profiles close in space and time indicate that float‐estimated *p*CO_2_ are consistent with the uncertainty of ±2.86% but that the float data may be biased high by ~4 μatm (Fay et al., 2018; Gray et al., 2018; Williams et al., 2018). While uncorrelated errors will average out given enough floats, a network‐wide bias could have a significant impact on derived CO_2_ flux estimates.

To test the impact of a systemic bias of this magnitude on our combined SOCCOM and SOCAT flux estimates, we recomputed the neural network and Jena interpolation scheme estimates using a SOCCOM product uniformly lowered by 4 μatm. This offset increased the calculated Southern Ocean carbon sink by a mean value of −0.15 Pg C/yr for the neural network and Jena CarboScope SOCAT+SOCCOM products and −0.21 Pg C/yr for the SOCCOM‐weighted products (Figure S4). While a possible offset of this magnitude would reduce the overall impact of the new SOCCOM float data, it is too small to eliminate the reduction in the Southern Ocean sink magnitude. Therefore, this test increases our confidence that the reduction in the air‐sea CO_2_ flux is not solely caused by a systematic measurement bias, but is indeed a robust signal detected by the SOCCOM floats. Naturally, if a more accurate determination of bias finds a larger difference between float‐estimated pCO_2_ and shipboard observations, this conclusion must be revisited.

An additional test of whether the SOCCOM and SOCAT data sets are compatible and whether introducing a 4‐μatm offset to the float *p*CO_2_ estimates improves their compatibility can be found by comparing the root mean square differences (RMSD) between the *p*CO_2_ fields estimated from the neural network and Jena CarboScope and the original observational products (Table S2). Unsurprisingly, the SOCAT‐only runs fit the individual SOCAT observations best, while the SOCCOM‐weighted runs fit the SOCCOM observations best. However, the combined SOCAT+SOCCOM product yielded RMSD values for both SOCAT and SOCCOM observations that were approximately equivalent to the fit yielded by the mapping products trained only with the respective individual data sets. This indicates that the data sets are consistent to within the ability of these mapping methods to recreate observed surface *p*CO_2_ and that the mapping products based on the combined data set yield a result that best explains both data sets. The largest RMSD values were calculated for the SOCAT‐only product compared to the SOCCOM data set and the SOCCOM‐weighted product compared to the SOCAT observations, indicating that the addition of SOCCOM observations does shift the mapped *p*CO_2_, consistent with the earlier analysis of the impact on air‐sea fluxes. Furthermore, decreasing the SOCCOM data uniformly by 4 μatm in the SOCAT+SOCCOM product did not yield a different RMSD than the original SOCAT+SOCCOM product, meaning that the fit of the mapped products to the observations cannot confirm or disprove the existence of a 4 μatm bias in the SOCCOM observations. One caveat is that we are not testing these previously published methods here and did not withhold data from the training data set when generating the SOCAT+SOCCOM solution. Therefore, this does not test if the neural network and Jena CarboScope have too much freedom to match the training data.

The SOM‐FFN is a global approach, with biogeochemical biomes distinguished by input properties but not explicitly by latitude. This means that the neural network output is influenced by SOCAT observations outside of the Southern Ocean, even in the SOCCOM‐weighted run. It is not straightforward to determine the exact influence of SOCAT versus SOCCOM observations, as the neural network derived relationships between input variables and *p*CO_2_ observations are nonlinear, and therefore do not scale based on the absolute number of observations. This is an additional reason for the SOCCOM‐weighted run as it establishes some idea of the method sensitivity to the local density of the two data sets. Additionally, the Jena CarboScope interpolation scheme is only affected by local observations within the a priori correlation radius, so the overall agreement between the two methods gives a strong indication that the 2015–2017 time period is heavily influenced by the SOCCOM floats.

The addition of float data starting in 2014 has some impact on prior years' flux estimates. For the neural network, addition of float data impacts two prior years, back through 2012. Prior to then, little difference is seen between the three data products, implying that the neural network establishes different relationships through time, likely due to increasing atmospheric *p*CO_2_, as there is no explicit time input to the neural network. The main impact on the Jena CarboScope method starts in 2014, though there is a smaller impact (~0.1 Pg C/yr) in earlier years due to its prescribed a priori correlation structure. Comparison of the *p*CO_2_ calculated from the mapping methods to the SOCCOM and SOCAT data sets indicates that the mapping methods are able to match the new data, even though the SOCCOM data are only present for the final four years of the data sets. Given that the number of SOCCOM float observations continues to increase and the difference between the SOCAT‐only and SOCAT+SOCCOM annual flux estimates has increased with each new year of SOCCOM float data, it appears that adding more float data increases the weight of the SOCCOM data set in the interpolation schemes. Therefore, the addition of more years of SOCCOM data may continue the divergence of the SOCAT‐only and SOCAT+SOCCOM products. In the next two sections we investigate how sampling differences between SOCAT and SOCCOM could impact estimates of the Southern Ocean flux.

### 3.2 SOCAT and SOCCOM Spatial and Temporal Sampling Differences

We use subsampled daily model output to explore the potential impact that the difference in sampling distribution of the SOCAT and SOCCOM data sets has on the calculated air‐sea carbon dioxide flux. While we do not know the true surface CO_2_ flux, high‐resolution models provide analogs to the real world that can be used to test whether the difference in sampling may account for some of the differences in air‐sea flux estimates using the two data sets.

**Figure 8:**
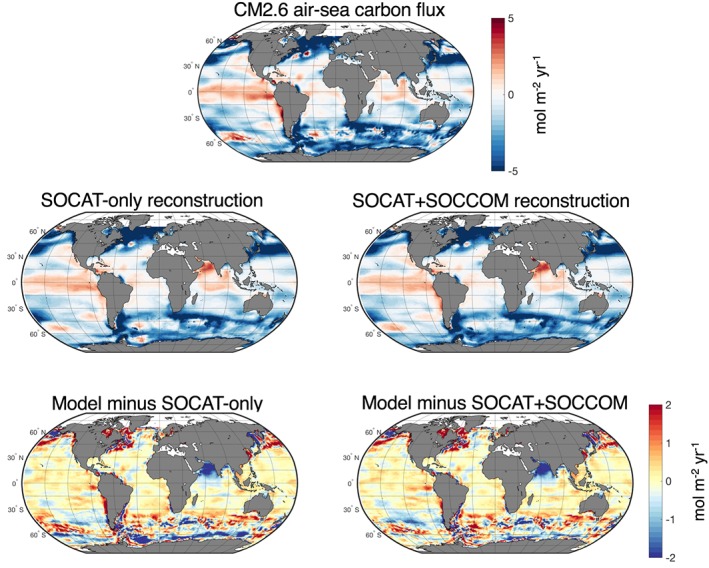
True model air‐sea flux, air sea fluxes based on neural network derived from SOCAT‐only and SOCAT+SOCCOM sample locations, and flux differences from model. Air‐sea fluxes were calculated in the same way for true model *p*CO_2_ and neural network mapped *p*CO_2_. The region with the most consistent differences between the neural network derived fluxes and model fluxes is the Southern Ocean. Addition of the float locations to the neural network decreases some of the large biases near Antarctica and reduces the root‐mean‐square differences between model and mapped *p*CO_2_ (Figure 9). SOCCOM = Southern Ocean Carbon and Climate Observations and Modeling; SOCAT = Surface Ocean CO_2_ Atlas v6.

**Figure 9:**
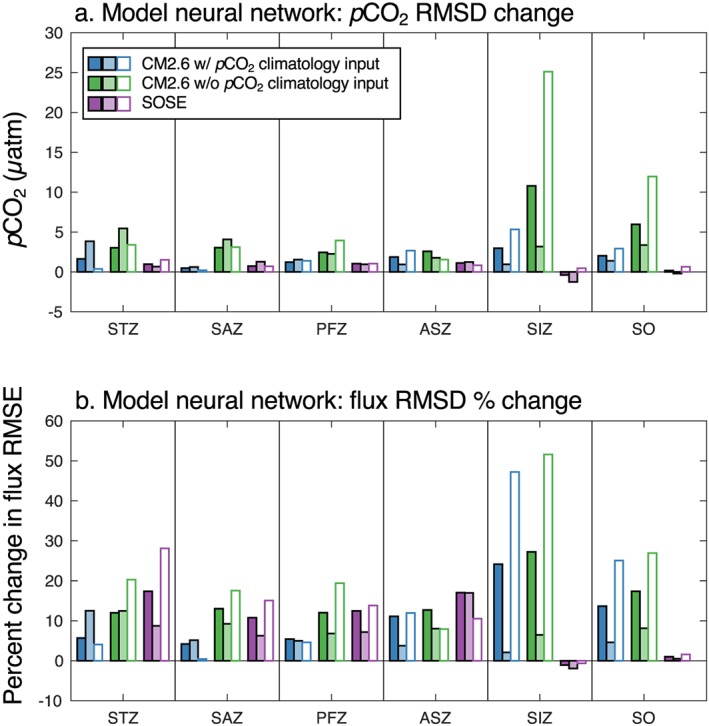
Model subsampling experiment with neural network results. (a) The difference between *p*CO_2_ RMSD for the SOCAT‐only and SOCAT+SOCCOM mapping of CM2.6 and SOSE (SOCAT‐only RMSD minus SOCAT+SOCCOM; positive values indicate an improvement in re‐creation of model output). (b) The percent improvement of neural network flux RMSD. For all cases, solid bars represent annual means, light bars represent summer (December–February), and outlined bars with no fill represent winter (June–August). The neural network approach for CM2.6 was run both with a climatological *p*CO_2_ input to the SOM based on model output and without any *p*CO_2_ climatology to demonstrate the sensitivity of the method to prior knowledge of the true seasonal cycle of *p*CO_2_. SOCCOM = Southern Ocean Carbon and Climate Observations and Modeling; SOCAT = Surface Ocean CO_2_ Atlas v6; STZ = Subtropical Zone; SAZ = Subantarctic Zone; PFZ = Polar Frontal Zone; ASZ = Antarctic‐Southern Zone; SIZ = Seasonal Ice Zone; RMSD = root‐mean‐square differences; RMSE = root‐mean‐square error.

Comparison of the estimated air‐sea CO_2_ flux using actual CM2.6 surface *p*CO_2_ output and fluxes with the neural network‐derived *p*CO_2_ indicates that the Southern Ocean is the basin with the highest differences in estimated fluxes (Figure 8). This conclusion is based on the CM2.6 case only, as SOSE output only covers south of ~29°S. Neural network results from the subsampled model output were evaluated based on the RMSD between mapped and true monthly *p*CO_2_ as a way to determine whether adding float observation locations decreased differences from true model output (Figure 9a). The percent change in air‐sea flux RMSD is also shown in order to understand the impact that changes in mapped *p*CO_2_ have on the flux estimates (Figure 9b). The differences between the SOCAT‐only and SOCAT+SOCCOM products are shown in Figures 9a and 9b; the SOCCOM‐weighted output had a broadly similar impact (actual model and neural network‐derived output differences for air‐sea fluxes, *p*CO_2_ RMSD, and air‐sea flux RMSD results shown in Tables S4–S6). When a *p*CO_2_ climatology was used in the SOM step of the neural network, the addition of SOCCOM float locations reduced the regional RMSD in CM2.6 output by 0.5 to 3 μatm (Figure 9a and Table S5). This is a somewhat small reduction relative to the mean RMSD across all regions of 28.5 μatm, but resulted in an average improvement of the flux RMSD of 10% (Figure 9b and Table S6). The impact on SOSE *p*CO_2_ RMSD was smaller, with a range of −0.49 μatm in the SIZ (indicating a worse reproduction of surface *p*CO_2_) to 1.1 μatm in the ASZ, and a mean impact of 0.7 μatm (Figure 9a). The smaller impact on SOSE output may indicate that the neural network is better able to constrain the single ocean basin in SOSE than the global ocean in CM2.6. When the *p*CO_2_ climatology was not used for the CM2.6 SOM biome creation, the *p*CO_2_ RMSD improvement increased to a mean annual impact of 4.4 μatm and a mean improvement in the flux estimate RMSD of 15% (Figures 9a and 9b). In the real‐world approach, use of the Takahashi et al. (2009) climatology in the neural network likely represents a midpoint between perfect knowledge of the seasonal cycle of *p*CO_2_ and no information; thus, these two CM2.6 reconstructions are shown as two possible endpoints.

The impact of adding SOCCOM locations on *p*CO_2_ RMSD is not consistently different for Summer (December–February) and winter (June–August; Figure 9a). However, the impact on flux was greater for wintertime in most regions, likely due to the seasonal asymmetry in wind speed (Figure 9b). The impact of float sampling on the annual flux is more variable, though with improvements to the returned flux with the addition of float locations in most cases. In the case of a perfect *p*CO_2_ climatology input to the SOM, both the CM2.6 SOCAT‐only and SOCAT+SOCCOM derived Southern Ocean fluxes were similar, with the SOCAT‐only uptake 0.22 Pg C/yr stronger than the model and the SOCAT+SOCCOM uptake 0.14 Pg C/yr stronger than the true model flux (Table S4). The SOCCOM‐weighted uptake was 0.29 Pg C/yr stronger than the model. For SOSE, the neural network‐derived SOCAT‐only Southern Ocean uptake was 0.38 Pg C/yr stronger than the true model, while the SOCAT+SOCCOM uptake was 0.26 Pg C/yr stronger than the model and the SOCCOM‐weighted uptake was 0.29 Pg C/yr stronger (Table S4). However, in the CM2.6 test where the SOM did not have the climatological *p*CO_2_ input, the SOCAT‐only uptake was 0.34 Pg C/yr weaker than the true model flux, while the SOCAT+SOCCOM derived flux and the SOCCOM‐weighted flux were within <0.1 Pg C/yr of the true model flux. These results indicate that the importance of adding wintertime observations is in part related to the quality of a priori information about the seasonal cycle of *p*CO_2_, which is likely worse in the Southern Ocean than in most other regions of the ocean. Furthermore, the approach used here primarily tested the impact of adding samples at the times and locations of float observations. All inputs to the neural network in this model subsampling approach represent perfect observations of the mean monthly values. In the real world there are gaps, uncertainties, and biases in this data, which would likely influence the value of new observations. It is also important to note that the air‐sea carbon dioxide fluxes in these models are not perfect representations of the actual world and that there are commonly issues with the seasonal cycle of high latitude carbon fluxes in global climate models (Anav et al., 2013), including in both of these models (Figure S5). The value of new wintertime data is likely a function of the importance of the wintertime flux to the total annual flux. This analysis shows that the neural network may have difficulty in reproducing Southern Ocean air‐sea fluxes and that adding year‐round float observations can improve the reproduction of those fluxes, with a potential impact of several tenths of one Pg C/yr.

### 3.3 Impact of Float Sampling Frequency on Reconstructed CO_2_ Fluxes

Profiling floats sample over multiple years, but it is important to determine whether the 10‐day sampling frequency of float profiles is adequate to reconstruct the mean annual CO_2_ flux. This is primarily important for derived estimates of the air‐sea flux from profiling floats not based on interpolation mapping methods. Previous studies have indicated that sampling CO_2_ fluxes at a 10‐day frequency, as is possible from Argo style profiling floats, is insufficient to capture an unbiased mean annual flux (Monteiro et al., 2015). If floats are missing important signals due to their 10‐day sampling, they could return a biased CO_2_ flux even with good spatiotemporal coverage.

**Figure 10:**
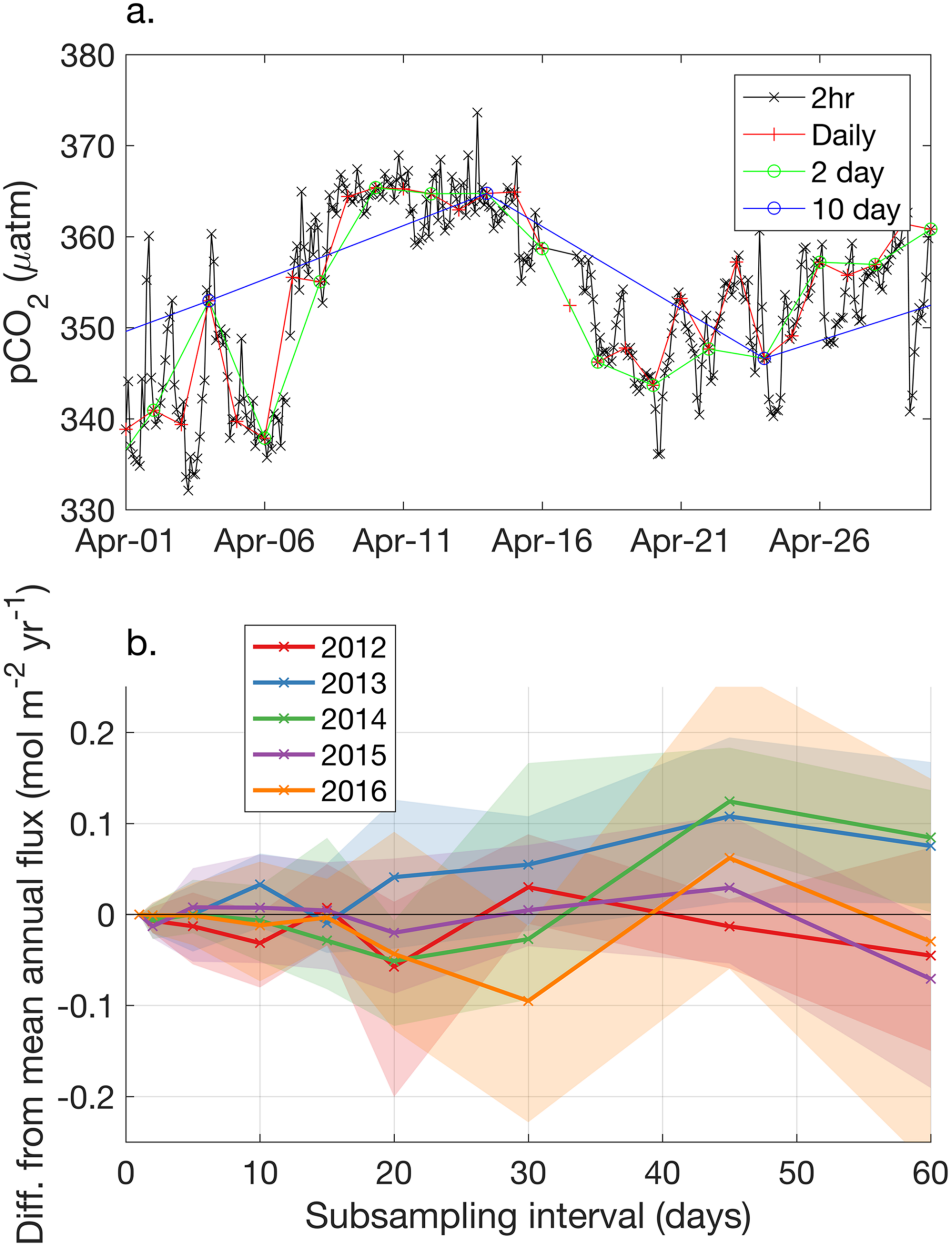
Example of *p*CO2 observations from a mooring off the coast of Tasmania (a) and the impact on the mean annual flux from subsampling *p*CO_2_ observations (b). (a) Observations of *p*CO_2_ made every 2 hr were subsampled at increasing intervals. Each subsampled record was then interpolated back to a daily time step, and air‐sea CO_2_ fluxes were calculated from daily atmospheric reanalysis data using the same method as for SOCCOM floats. The difference between mean annual fluxes from daily and subsampled records are plotted for 5 years of data (b), with the shaded areas representing the range of 12 analysis iterations (i.e., starting the subsampling on a different 2‐hr time step). Float observations are made once every 10 days, which should bias the mean annual flux by less than ±0.1 mol·m^−2^·yr^−1^ (<0.1 Pg C/yr over the entire Southern Ocean). SOCCOM = Southern Ocean Carbon and Climate Observations and Modeling.

The ability of profiling floats to capture annual air‐sea carbon dioxide fluxes with a 10‐day sampling frequency is assessed using high‐frequency mooring observations. However, instead of considering aliasing from interpolation of CO_2_ flux or the air‐sea *p*CO_2_ difference, as in Monteiro et al. (2015), here we interpolate only the oceanic *p*CO_2_ signal and then subsequently calculate fluxes from this time series. This avoids undersampling the highly variable and nonnormally distributed wind speed and sea level pressure fields and the subsequent nonlinear response of air‐sea CO_2_ fluxes and is consistent with the flux calculation method for the nonmapped float method in this study and in Gray et al. (2018). Mooring observations of *p*CO_2_ every 2 hr over 5 years from the Integrated Marine Observing System Maria Island National Reference Station Mooring at 42.6°S, 148.2°E off the coast of Tasmania (https://portal.aodn.org.au/) were subsampled at decreasing frequency, from once a day to every 60 days (Figure 10a). Daily values of *p*CO_2_ were interpolated from each of the subsampled data records. Air‐sea carbon dioxide fluxes were then calculated from the resulting daily values using the Wanninkhof (2014) air‐sea flux parameterization and the same atmospheric parameters described in section 2.3. This subsampling, interpolation, and subsequent flux calculation was performed 12 times by starting each iteration on a different 2‐hr sample.

Annual fluxes calculated from the 2‐day and less frequently subsampled data sets were compared to the annual flux calculated from the daily subsampled *p*CO_2_ record (Figure 10b). At intervals of less than 20 days, the difference in the mean annual flux was less than 0.1 mol m^‐2^/yr. If such a difference were present over the entire Southern Ocean, the equivalent impact would be <0.1 Pg C/yr. While the temporal variance of *p*CO_2_ at this one mooring is not likely to exactly match that of the Southern Ocean as a whole, this does indicate that the float sampling protocol of 10‐day intervals is unlikely to be a primary source of uncertainty in recreating the annual flux. Essentially, these results suggest that float observations made every 10 days do return an unbiased *p*CO_2_ estimate to within <0.1 Pg C/yr. Variability in wind speed and sea level pressure are significant contributors to the air‐sea CO_2_ flux variance, but in the flux calculation described in section 2.3 we use 6‐hr output from reanalysis products. Calculating air‐sea fluxes at a high‐resolution time step using matching atmospheric data captures much of the impact of high frequency atmospheric variability and allows a more robust estimate of the annual mean flux.

### 3.4 Implications for the Global Carbon Cycle

The combined SOCAT+SOCCOM product yields a Southern Ocean sink that is 0.4 Pg C/yr weaker over 2015–2017 than that calculated from shipboard data alone, approximately 1/3 of the total ship‐only derived Southern Ocean uptake of −1.14 ± 0.19 Pg C/yr (Figure 3). This impact is calculated from only two interpolation methods, so it is important to understand how these methods compare with prior estimates of Southern Ocean carbon uptake. In the current study we primarily consider the entire Southern Ocean, south of 35°S, but for ease of comparison with prior studies we also present fluxes south of 44°S in Table 1. Estimates of Southern Ocean carbon dioxide uptake during the 1990s and 2000s from a mix of ocean inversions, atmospheric inversions, ocean models, and surface observations yield a Southern Ocean flux south of 44°S of between −0.27 to −0.42 Pg C/yr (Gruber et al., 2009; Lenton et al., 2013; Table 1). This is consistent with uptake calculated from the two methods used in this study, with the Landschützer et al. (2016) method yielding an uptake of −0.24 ± 0.39 Pg C/yr and the Rödenbeck et al. (2013) approach an uptake of −0.44 ± 0.4 Pg C/yr for the same time period and geographic range. As noted earlier, the Southern Ocean uptake increased after the early 2000s. For 2015–2017, south of 44°S, the SOCAT‐only neural network derived air‐sea flux is −0.41 ± 0.15 and the Jena CarboScope‐based flux is −0.63 ± 0.17 Pg C/yr (Table 1). In contrast, the SOCAT+SOCCOM estimate for 2015–2017, south of 44°S, is −0.16 ± 0.18 Pg C/yr. Taken together, this implies that the impact to the Southern Ocean flux from the addition of SOCCOM float data likely represents a change in the mean air‐sea flux due to the inclusion of data from previously under sampled regions and seasons, and is not simply a method‐dependent result.

A long‐term mean anthropogenic flux into the Southern Ocean of ~1.1 Pg C/yr has been calculated from interior ocean measurements (Devries, 2014). If the contemporary Southern Ocean uptake is reduced, this implies either an error in the anthropogenic estimate or, more likely, an increase in the less well‐constrained natural carbon flux between the ocean and atmosphere.

**Figure 11:**
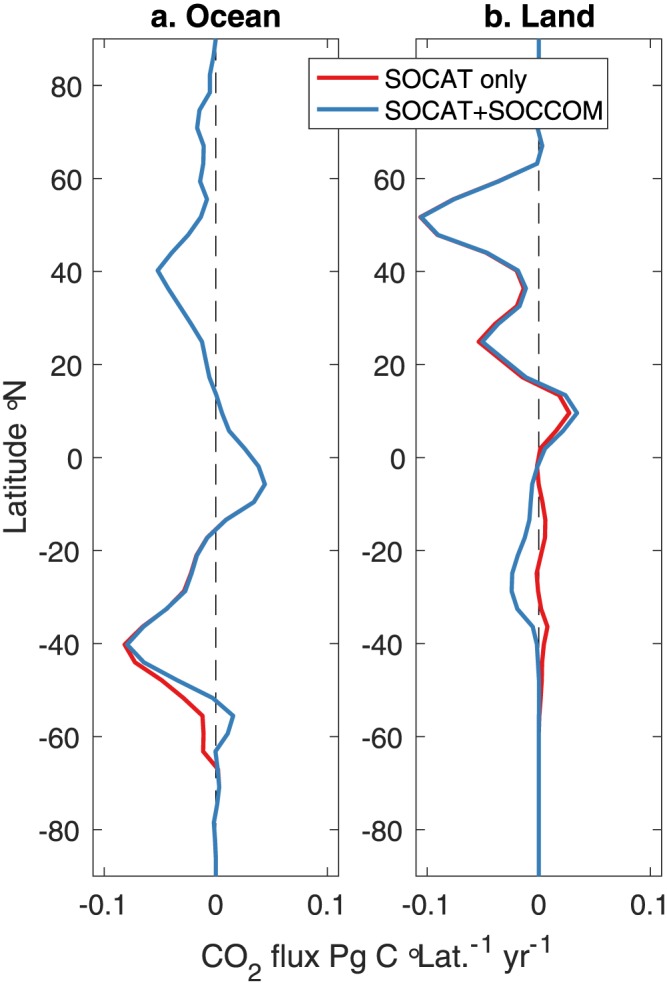
Mean atmospheric inversion derived land carbon fluxes for 2015–2017. Global ocean CO_2_ flux for SOCAT only and SOCAT+SOCCOM products based on the Jena CarboScope interpolation output (a) and land CO_2_ fluxes (b) calculated from the Jena CarboScope atmospheric inversion (positive to the atmosphere). The atmospheric inversion uses the oceanic fluxes as a fixed boundary condition, so this is primarily a test of the needed change in land CO_2_ fluxes to balance a change in the Southern Ocean flux. The difference indicates that compensating fluxes in the land or ocean would have a northern limit based on the atmospheric transfer model of ~5°S. SOCCOM = Southern Ocean Carbon and Climate Observations and Modeling; SOCAT = Surface Ocean CO_2_ Atlas v6.

Because the atmosphere is relatively well mixed and the atmospheric inventory of carbon is well constrained, a change in one region of the contemporary oceanic sink must be compensated for by a change in another ocean area or in the land carbon sink. The land carbon sink is poorly constrained and in the past was often calculated as the residual between the atmospheric increase and ocean uptake for given anthropogenic emissions (Peylin et al., 2013). To understand what region of the world might contain a compensating sink to offset a change in the Southern Ocean contemporary estimate, we used the Jena interpolated oceanic carbon fluxes for the SOCAT‐only and SOCAT+SOCCOM cases, respectively, as a fixed ocean prior in the Jena CarboScope atmospheric inversion (method details in Rödenbeck et al., 2003; Rödenbeck, 2005; Figure 11 and Table S7). This inversion uses atmospheric observations and an atmospheric transport model to effectively adjust the land sink for differences in the ocean sink. Due to strong zonal mixing in atmospheric transport, the land sink changes between the SOCAT‐only and SOCAT+SOCCOM products are limited to south of ~5°S (Figure 11). By construction, the inversion method used here takes the ocean CO_2_ flux as a fixed input, such that the decrease in the ocean uptake of carbon is offset by an increase in the land uptake. Alternatively, the change south of 35°S could also be balanced by a change in the poorly sampled Southern Hemisphere subtropical ocean carbon flux, or a combination of the two.

A reduced Southern Ocean contemporary carbon sink that is balanced by an increase in the Southern Hemisphere land sink (or a reduction in the carbon flux from land to atmosphere) is consistent, both in direction and magnitude, with a recent study that reassessed the global carbon cycle using heat‐based constraints on ocean carbon transport (Resplandy et al., 2018). That study suggested that the mean 1990–2010 contemporary air‐sea flux south of 20°S was reduced from 1.41 to 1.20 Pg C/yr, based on a change in the natural carbon flux from −0.21 to ~0 Pg C/yr. This was balanced in part by a reduction in Southern Hemisphere land outgassing of 0.24 Pg C/yr. Calculating the comparable change from our study in the contemporary ocean carbon flux south of 20°S yields a change of 0.54 Pg C/yr (Table S7). Our adjustment is somewhat larger, which could reflect that it represents a 3‐year period rather than a 20‐year mean. The agreement in sign supports our reduction in the estimated contemporary Southern Ocean carbon flux and points toward the change being primarily due to an increase in the underlying natural carbon outgassing. An alternative to changing the land outgassing of carbon is that a significant portion of the Southern Hemisphere ocean from 20°S to 35°S remains undersampled, as large regions of the Southern subtropical gyres and boundary regions, for example, along the South American coasts, are poorly sampled by both ships and floats. Increases in observations in these areas are needed to more accurately understand the partitioning between the subtropical ocean and Southern Hemisphere land.

## 4 Conclusions

In this study, we have shown that combining shipboard measurements and float‐derived estimates of *p*CO_2_ using two observation‐based methods for mapping and calculating the global carbon flux leads to a 0.4 Pg C/yr change in the estimated Southern Ocean carbon uptake, from ‐1.14 ± 0.19 Pg C/yr in the ship‐only estimate to a −0.75 ± 0.22 Pg C/yr uptake in the combined estimate. This is consistent with a recent float‐only estimate (Gray et al., 2018), though with a reduced magnitude. A portion of this impact may be explained by temporal and spatial sampling differences between SOCCOM profiling floats and the SOCAT observational record. At current observational densities, it appears that the shipboard data set may not fully constrain the magnitude of the mapped *p*CO_2_‐based flux estimates in recent years; we therefore recommend that a combined float and ship *p*CO_2_ product be used for future estimates of the global carbon flux. Such a product, like the one presented here, benefits from the high accuracy, long record, and global coverage of the shipboard data and the expanded spatial and seasonal coverage of the profiling floats. Using this product we find a reduction in the contemporary Southern Ocean carbon sink from two complementary mapping methods, which is potentially due to an adjustment of the natural carbon flux in this region and could be balanced by a reduction in the Southern Hemisphere land carbon source, consistent with another recent study based on independent data (Resplandy et al., 2018).

The SOCCOM data set has annual observations spread throughout the Southern Ocean, but contains estimates of *p*CO_2_ that at the moment have significant theoretical uncertainty (Williams et al., 2017). We tested the impact of a possible bias in float‐derived estimates of *p*CO_2_ and found that if it exists, such a systematic offset would reduce but not eliminate the impact of the new float *p*CO_2_ estimates. Therefore, we recommend further work to understand and correct for any bias in these new observations and to empirically determine the in situ accuracy of float‐derived *p*CO_2_. We also found that the 10‐day profiling frequency of SOCCOM floats is sufficient to capture the *p*CO_2_ variability necessary to reconstruct a mean annual flux. Despite nonnegligible uncertainty, the addition of profiling float observations to the Southern Ocean carbon budget has greatly improved our understanding of the CO_2_ fluxes during the past several years. A question that remains is whether the new fluxes that result from combining the SOCCOM observations with the SOCAT data set reflect a difference in the mean state of the Southern Ocean, additional variability around the mean, or a combination of the two. Work exploring decadal‐scale variability in the Southern Ocean carbon flux (Gruber, Landschützer, & Lovenduski, 2019; Keppler & Landschützer, 2019; Landschützer et al., 2015), recent reduction in Antarctic sea ice cover (Schlosser et al., 2018; Stuecker et al., 2017), and changes in sea surface temperature (Blunden & Arndt, 2017) and salinity (Haumann et al., 2016) all indicate a highly variable system on multiple scales. The impact of adding float observations to our Southern Ocean carbon flux estimates is on the order of previously inferred interannual variability, suggesting that we need sustained year‐round observations across much of the region in order to fully understand the Southern Ocean's role in the global carbon cycle.

## Supporting information



Supporting Information S1
