# Supplementary material for: Reassessing Southern Ocean Air‐Sea CO2 Flux Estimates With the Addition of Biogeochemical Float Observations
Source: Global Biogeochem Cycles. 2019 Nov 16;33(11):1370–88. doi: 10.1029/2019GB006176 (PMC6988491; doi:10.1029/2019GB006176)
Supplement: Supplementary file 1 — Supporting Information S1 [file GBC-33-1370-s001.docx]

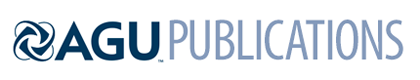


*Global Biogeochemical Cycles*

Supporting Information for

**Reassessing Southern Ocean air-sea CO_2_ flux estimates with the addition of biogeochemical float observations**

Seth M. Bushinsky^1^, Peter Landschützer^2^, Christian Rödenbeck^3^, Alison R. Gray^4^, David Baker^5^, Matthew R. Mazloff^6^, Laure Resplandy^7^, Kenneth S. Johnson^8^, Jorge L. Sarmiento^1^

^1^Program in Atmospheric and Oceanic Sciences, Princeton University, Princeton NJ, USA; ^2^Max Planck Institute for Meteorology, Hamburg, Germany; ^3^Max Planck Institute for Biogeochemistry, Jena, Germany; ^4^School of Oceanography, University of Washington, Seattle, WA, USA; ^5^Cooperative Institute for Research in the Atmosphere, Colorado State University, Fort Collins CO, USA ; ^6^Scripps Institution of Oceanography, University of California San Diego, La Jolla CA, USA; ^7^Department of Geosciences and Princeton Environmental Institute, Princeton University, Princeton NJ, USA ; ^8^Monterey Bay Aquarium Research Institute, Moss Landing, CA, USA

**Contents of this file**

Text S1 to S4

Figures S1 to S5

Tables S1 to S7

**Introduction**

Included in the Supporting Information are a table listing SOCCOM profiling floats used in this work, figures for the Jena CarboScope interpolation scheme that are equivalent to those presented in the main text for the neural network, a sensitivity test of the effect of introducing a 4 µatm adjustment to the SOCCOM *p*CO_2_ estimates, additional details from the modeling subsampling analysis, tables showing results from the model subsampling approach, and a table showing results from the atmospheric inversion analysis.

**Text S1. Jena CarboScope interpolation scheme difference plots**

Annual, winter, and summer mean fluxes for the Jena CarboScope interpolation scheme are shown in Figure S1. Monthly mean differences plots for the three *p*CO_2_ datasets are presented for the Jena CarboScope interpolation scheme (Figures S2 and S3). The overall location and magnitude of differences in air-sea fluxes between the SOCAT only and SOCCOM only runs are similar, though the Jena CarboScope has a stronger response to the addition of float observations than it does to the removal of shipboard data

**Text S2. Sensitivity test to a possible float *p*CO_2_ bias**

Comparisons of float *p*CO_2_ estimates to shipboard measurements have a large spread, but indicate a possible float bias of <4 µatm *p*CO_2_ higher than shipboard observations. To test the impact of a bias of this magnitude we calculated SOCAT+SOCCOM runs with the SOCCOM *p*CO­_2_ estimates artificially lowered by 4 µatm (SOCAT+SOCCOM minus 4) for both the neural network and the Jena CarboScope interpolation. We also performed a SOCCOM only run offset by 4 µatm (SOCCOM only minus 4) for the neural network alone. An offset of that magnitude reduces the magnitude of the floats’ impact (Figure S4), but does not eliminate the reduction in the Southern Ocean carbon sink. The RMSD between the SOCAT and SOCCOM observations and each *p*CO_2_ product indicates that the SOCAT+SOCCOM and SOCAT+SOCCOM minus 4 runs explain the two datasets equally well, while the SOCAT only and SOCCOM only runs are better at explaining their input observations (Table S2).

**Text S3. Model subsampling**

Examples of the seasonal cycle of air-sea CO_2_ fluxes in two regions of the Southern Ocean indicate the difficulties in using model output to represent the Southern Ocean CO_2_ flux (Figure S5). In the STZ, the seasonal cycle of CM2.6 and SOSE are in similar phase and magnitude to the direct float estimates of the monthly mean flux. However, in the ASZ, CM2.6 is anti-correlated in phase to the float estimates, while SOSE is near zero throughout the year.

**Text S4. Southern Hemisphere (90°S – 20°S) CO_2_ flux budget**

Using the Jena CarboScope ocean interpolation scheme and atmospheric inversion and an assumed constant anthropogenic carbon uptake of -1.2 Pg C yr^-1^ from 90°S to 20°S, we calculate an adjustment in the natural carbon flux of ~0.55 Pg C yr^-1^. This is due to a reduction in the contemporary carbon flux due to inclusion of the SOCCOM observations.

**Figure S1. Mean 2015-2017 summer, winter, and annual Southern Ocean fluxes from all three *p*CO_2_ products.** Jena interpolation scheme fluxes show similar patterns to the network fluxes in Figure 4. Addition of the float data strongly impacts winter (May – Oct.) fluxes around the Polar Front in both the SOCAT+SOCCOM and SOCCOM-weighted products. Summer (Nov. – Apr.) fluxes in the SOCCOM-weighted product indicate more outgassing than in the other two products. Overall, addition of float data decreases the mean annual uptake.

**Figure S2. Impact of adding SOCCOM floats on the Jena interpolation scheme air-sea CO_2_ fluxes.** Monthly mean differences between Jena interpolation scheme, SOCAT and SOCCOM minus SOCAT only.

**Figure S3. Impact of removing SOCAT observations on the Jena interpolation scheme air-sea CO_2_ fluxes.** Monthly mean differences between Jena interpolation scheme SOCCOM only minus SOCAT + SOCCOM.

**Figure S4. Sensitivity of float neural network products to possible bias in the float *p*CO_2_ product.** To test the sensitivity of the interpolation mapping methods that included SOCCOM float data, two new runs were conducted with all float *p*CO_2_ lowered by 4 µatm (open symbols). This increased the calculated Southern Ocean (<35°S) carbon uptake but did not eliminate the difference between the SOCAT+SOCCOM and SOCAT-only products or the large difference between those products and the SOCCOM-weighted product. All symbols represent the mean of the neural network and Jena CarboScope output.


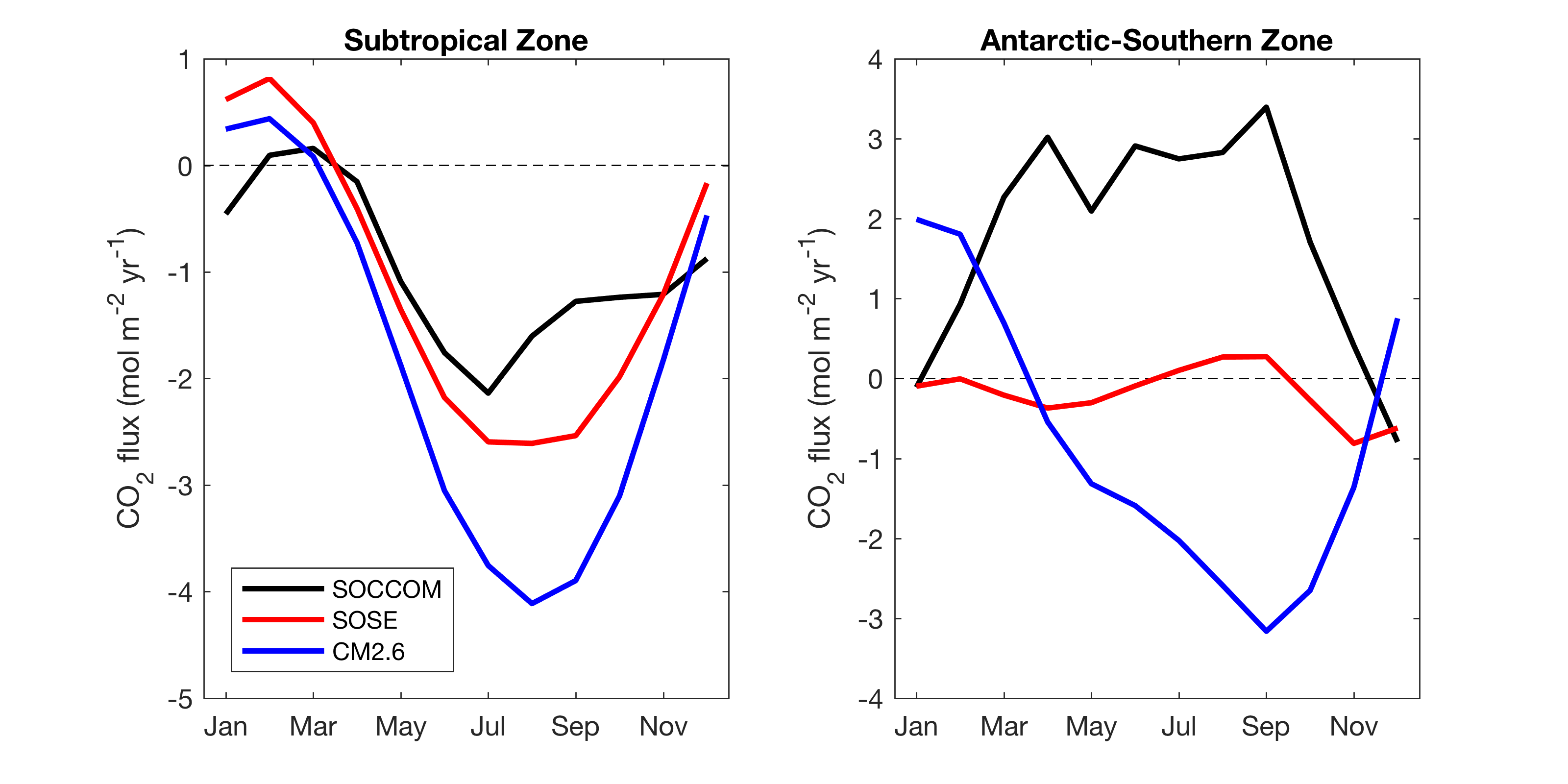


**Figure S5**. Seasonal CO_2_ flux comparisons in two example regions. Monthly mean fluxes were calculated for the Subtropical Zone and the Antarctic-Southern Zone for the non-mapped float *p*CO_2_ estimates, SOSE, and CM2.6. The models agree in the phase of the seasonal cycle in the STZ, though with a significant difference in the wintertime uptake between observations and CM2.6. In the ASZ the floats indicate much stronger outgassing in the wintertime than SOSE, which is neutral, or CM2.6, which indicates a strong uptake.

**Table S1.** **Identification numbers (WMO and UW), date and location of first profile, and the number of profiles (n) completed during the study period ending December 31, 2017 for all SOCCOM floats used in the present study.**

| **WMO ID** | **UW ID** | **Date of first** | **Dep. Latitude** | **Dep. longitude** | **n** | **WMO ID** | **UW ID** | **Date of first** | **Dep. Latitude** | **Dep. longitude** | **n** |  |
| --- | --- | --- | --- | --- | --- | --- | --- | --- | --- | --- | --- | --- |
| 5905131 | 12733 | 9-Oct-17 | -34.633 | 4.191 | 9 | 5905069 | 12558 | 12-Jan-17 | -54.816 | 95.574 | 36 |  |
| 5904685 | 9642 | 9-Jan-17 | -39.002 | -103.114 | 36 | 5904396 | 9031 | 12-Apr-14 | -55.01 | -150.011 | 130 |  |
| 5904675 | 9749 | 13-Jan-16 | -39.451 | 99.47 | 70 | 5904657 | 9662 | 10-Jan-16 | -56.48 | -57.196 | 70 |  |
| 5904395 | 9254 | 21-Apr-14 | -39.698 | -150.019 | 172 | 5905076 | 12386 | 22-Feb-17 | -57.178 | -126.307 | 32 |  |
| 5904674 | 9630 | 31-May-16 | -39.964 | -172.569 | 57 | 5904854 | 12575 | 28-Dec-16 | -57.349 | -68.005 | 37 |  |
| 5905134 | 12723 | 19-Sep-17 | -42.98 | -9.927 | 11 | 5904695 | 9265 | 14-May-16 | -57.981 | -169.951 | 58 |  |
| 5904474 | 9313 | 7-Dec-14 | -44.602 | 7.197 | 127 | 5904658 | 9655 | 9-Jan-16 | -57.987 | -56.503 | 70 |  |
| 5904187 | 9101 | 18-Apr-14 | -44.977 | -150.151 | 171 | 5904982 | 12573 | 28-Dec-16 | -59.21 | -68.508 | 37 |  |
| 5904841 | 12382 | 12-Jan-17 | -45.007 | -102.991 | 36 | 5904659 | 9657 | 8-Jan-16 | -59.635 | -55.38 | 70 |  |
| 5904765 | 9762 | 31-May-16 | -46.653 | -171.541 | 57 | 5905099 | 12388 | 16-Apr-17 | -59.986 | 170.174 | 26 |  |
| 5904470 | 8514 | 26-Mar-15 | -47.025 | 143.871 | 96 | 5904185 | 9092 | 7-Apr-14 | -60 | -149.928 | 133 |  |
| 5904683 | 9650 | 3-Mar-16 | -47.089 | 93.079 | 65 | 5904660 | 9652 | 7-Jan-16 | -60.623 | -54.63 | 70 |  |
| 5905135 | 12742 | 21-Sep-17 | -47.498 | -13.083 | 11 | 5904397 | 9125 | 22-Jan-15 | -61.022 | -0.029 | 105 |  |
| 5904693 | 9634 | 20-May-16 | -47.997 | -169.974 | 57 | 5905367 | 12755 | 28-Dec-17 | -63.027 | 69.943 | 1 |  |
| 5904473 | 9260 | 28-Jan-15 | -49.013 | 12.964 | 67 | 5904184 | 9091 | 3-Apr-14 | -63.973 | -150.035 | 133 |  |
| 5904679 | 9757 | 19-Jan-16 | -49.765 | 78.823 | 69 | 5904856 | 12545 | 30-Dec-16 | -64.14 | -69.096 | 37 |  |
| 5904188 | 9095 | 15-Apr-14 | -49.932 | -149.89 | 152 | 5905077 | 12379 | 18-Feb-17 | -65.013 | -125.899 | 32 |  |
| 5904763 | 9632 | 19-May-16 | -49.963 | -169.775 | 58 | 5904468 | 9099 | 19-Jan-15 | -66.002 | 0.058 | 105 |  |
| 5904677 | 9631 | 1-Apr-16 | -50.48 | 147.181 | 68 | 5905078 | 12371 | 17-Feb-17 | -66.389 | -126.083 | 32 |  |
| 5904682 | 9637 | 2-Mar-16 | -50.597 | 90.399 | 65 | 5904980 | 12543 | 2-Jan-17 | -66.423 | -74.232 | 36 |  |
| 5904842 | 12552 | 16-Jan-17 | -50.995 | -103.095 | 35 | 5904471 | 9094 | 21-Dec-14 | -66.945 | 359.968 | 108 |  |
| 5904761 | 9660 | 18-May-16 | -51.923 | -169.911 | 58 | 5905075 | 8501 | 25-Jan-17 | -67.005 | -102.999 | 34 |  |
| 5904678 | 9744 | 7-Apr-16 | -51.952 | 148.666 | 69 | 5904472 | 9275 | 18-Jan-15 | -67.661 | -1.762 | 105 |  |
| 5904186 | 9018 | 13-Apr-14 | -52.544 | -149.971 | 172 | 5904981 | 12390 | 9-Jan-17 | -68.257 | -128.458 | 34 |  |
| 5904684 | 9602 | 28-Feb-16 | -52.973 | 87.67 | 62 | 5905102 | 12363 | 19-Apr-17 | -68.568 | 172.059 | 15 |  |
| 5904663 | 9668 | 9-Dec-15 | -53.328 | -80.296 | 73 | 5904860 | 12541 | 12-Jan-17 | -72.355 | -146.349 | 36 |  |
| 5904469 | 9096 | 11-Dec-14 | -53.513 | 0.015 | 109 | 5904857 | 12381 | 13-Jan-17 | -75.647 | -156.978 | 36 |  |
| 5905079 | 12542 | 23-Feb-17 | -53.869 | -125.677 | 31 |  |  |  |  |  |  |  |
| 5905370 | 12734 | 24-Dec-17 | -54.024 | 68.027 | 1 |  |  |  |  |  |  |  |
| 5904661 | 9646 | 29-Dec-15 | -54.258 | -89.251 | 71 |  |  |  |  |  |  |  |
| 5904662 | 9666 | 29-Dec-15 | -54.262 | -89.253 | 75 |  |  |  |  |  |  |  |
|  |  |  |  |  |  |  |  |  |  |  |  |  |
|  |  |  |  |  |  |  |  |  |  |  |  |  |
|  |  |  |  |  |  |  |  |  |  |  |  |  |
|  |  |  |  |  |  |  |  |  |  |  |  |  |

**Table S2. Root-mean-square differences (RMSD) between observational datasets and mapping products south of 35°S, 2015-2017.**

|  | | |  |  | |  | |
| --- | --- | --- | --- | --- | --- | --- | --- |
|  | RMSD to SOCAT | |  | RMSD to SOCCOM | | |  |
| (µatm) | NN^a^ | JCS^b^ |  | NN^a^ | JCS^b^ | |  |
| SOCAT-only | 16 | 33 |  | 26 | 29 | |  |
| SOCAT+SOCCOM | 16 | 33 |  | 23 | 18 | |  |
| SOCCOM-weighted | 22 | 41 |  | 23 | 17 | |  |
| SOCAT+SOCCOM minus 4 µatm | 16 | 33 |  | 23 | 18 | |  |

^a^SOCAT observations were gridded first to 1°x1° bins to mimic the dataset used for the neural network (NN) training.

^b^Jena CarboScope RMSD values were calculated using the unbinned SOCAT observations.

**Table S3. Mean annual Southern Ocean (south of 35°S) carbon fluxes from the Jena CarboScope and neural network mapping methods**

| **Southern Ocean**  **(Pg C yr^-1^)** | **SOCAT-only** | | **SOCAT+SOCCOM** | |  | **SOCCOM-weighted** | |
| --- | --- | --- | --- | --- | --- | --- | --- |
|  | **NN** | **Jena** | **NN** | **Jena** |  | **NN** | **Jena** |
| 2014 | -1.08 | -1.44 | -0.98 | -1.36 |  | -0.66 | -1.07 |
| 2015 | -1.07 | -1.11 | -0.89 | -0.77 |  | -0.38 | -0.56 |
| 2016 | -1.10 | -1.40 | -0.83 | -0.80 |  | -0.24 | -0.40 |
| 2017 | -1.12 | -1.05 | -0.83 | -0.39 |  | -0.19 | -0.33 |

**Table S4. Neural network mapped model air-sea fluxes minus actual model fluxes**

| Flux (Pg C yr^-1^) | | STZ | SAZ | PFZ | ASZ | SIZ | SO (south of 35°S) |
| --- | --- | --- | --- | --- | --- | --- | --- |
| CM2.6 w/ pCO_2_ climatology input | SOCAT-only | 0.06 | -0.04 | -0.02 | -0.04 | -0.17 | -0.22 |
|  | SOCAT+SOCCOM | 0.02 | -0.09 | -0.01 | 0.01 | -0.07 | -0.14 |
|  | SOCCOM-weighted | -0.02 | -0.09 | -0.04 | -0.01 | -0.14 | -0.29 |
|  |  |  |  |  |  |  |  |
| CM2.6 w/o pCO_2_ climatology | SOCAT-only | 0.11 | 0.03 | 0.10 | 0.11 | 0.00 | 0.34 |
|  | SOCAT+SOCCOM | 0.04 | -0.08 | 0.04 | 0.06 | 0.02 | 0.07 |
|  | SOCCOM-weighted | 0.00 | -0.08 | 0.00 | 0.00 | 0.01 | -0.06 |
|  |  |  |  |  |  |  |  |
| SOSE | SOCAT-only | -0.07 | -0.10 | -0.06 | 0.02 | -0.17 | -0.38 |
|  | SOCAT+SOCCOM | -0.03 | -0.03 | -0.03 | 0.00 | -0.18 | -0.26 |
|  | SOCCOM-weighted | -0.03 | -0.03 | -0.03 | -0.01 | -0.18 | -0.29 |

**Table S5. Neural network mapped model output *p*CO_2_ RMSD**

|  |  | STZ |  |  | SAZ |  |  | PFZ |  |  | ASZ |  |  | SIZ |  |  | SO (south of 35°S) | | |
| --- | --- | --- | --- | --- | --- | --- | --- | --- | --- | --- | --- | --- | --- | --- | --- | --- | --- | --- | --- |
| *p*CO_2_ RMSD (µatm) | | All | DJF | JJA | All | DJF | JJA | All | DJF | JJA | All | DJF | JJA | All | DJF | JJA | All | DJF | JJA |
| CM2.6 w/ *p*CO_2_ climatology input | SOCAT-only | 23.1 | 30.0 | 17.9 | 29.8 | 35.5 | 25.0 | 20.9 | 24.7 | 18.3 | 18.1 | 21.3 | 19.8 | 50.8 | 40.6 | 60.9 | 35.6 | 33.4 | 38.9 |
|  | SOCAT+SOCCOM | 21.5 | 26.2 | 17.5 | 29.3 | 34.9 | 24.8 | 19.6 | 23.2 | 17.0 | 16.3 | 20.3 | 17.1 | 47.8 | 39.6 | 55.6 | 33.6 | 32.0 | 35.9 |
|  | SOCCOM-weighted | 22.5 | 27.0 | 18.8 | 28.9 | 34.6 | 24.5 | 19.7 | 23.6 | 17.4 | 16.1 | 21.0 | 15.3 | 53.9 | 43.5 | 66.2 | 36.5 | 33.7 | 41.2 |
|  |  |  |  |  |  |  |  |  |  |  |  |  |  |  |  |  |  |  |  |
| CM2.6 w/o *p*CO_2_ climatology | SOCAT-only | 27.1 | 35.6 | 22.6 | 40.3 | 43.6 | 38.7 | 23.9 | 26.5 | 23.2 | 21.3 | 21.3 | 23.7 | 52.1 | 70.8 | 52.6 | 39.0 | 49.8 | 38.2 |
|  | SOCAT+SOCCOM | 24.0 | 30.1 | 19.2 | 37.3 | 39.5 | 35.6 | 21.5 | 24.3 | 19.3 | 18.7 | 19.5 | 22.1 | 41.3 | 67.6 | 27.5 | 33.0 | 46.4 | 26.3 |
|  | SOCCOM-weighted | 25.5 | 29.9 | 22.3 | 37.4 | 39.9 | 36.4 | 22.0 | 25.1 | 20.4 | 18.4 | 20.7 | 21.1 | 43.4 | 73.6 | 26.5 | 34.2 | 49.5 | 26.5 |
|  |  |  |  |  |  |  |  |  |  |  |  |  |  |  |  |  |  |  |  |
| SOSE | SOCAT-only | 32.8 | 33.5 | 32.6 | 32.4 | 33.0 | 32.3 | 15.7 | 16.4 | 15.3 | 7.9 | 8.0 | 7.8 | 59.2 | 58.9 | 60.3 | 39.9 | 40.0 | 40.4 |
|  | SOCAT+SOCCOM | 31.8 | 32.9 | 31.1 | 31.7 | 31.7 | 31.6 | 14.6 | 15.4 | 14.3 | 6.7 | 6.8 | 6.9 | 59.6 | 60.2 | 59.9 | 39.8 | 40.2 | 39.7 |
|  | SOCCOM-weighted | 30.9 | 32.0 | 30.6 | 32.3 | 32.5 | 32.1 | 14.8 | 15.7 | 14.2 | 7.0 | 7.5 | 7.0 | 56.8 | 58.5 | 55.3 | 38.4 | 39.4 | 37.5 |

**Table S6. Neural network mapped model output air-sea flux RMSD**

|  |  | STZ |  |  | SAZ |  |  | PFZ |  |  | ASZ |  |  | SIZ |  |  | SO (south of 35°S) | | |
| --- | --- | --- | --- | --- | --- | --- | --- | --- | --- | --- | --- | --- | --- | --- | --- | --- | --- | --- | --- |
| Flux RMSD (Pg C yr^-1^) | | All | DJF | JJA | All | DJF | JJA | All | DJF | JJA | All | DJF | JJA | All | DJF | JJA | All | DJF | JJA |
| CM2.6 w/ *p*CO_2_ climatology input | SOCAT-only | 0.44 | 0.46 | 0.47 | 0.59 | 0.56 | 0.61 | 0.46 | 0.41 | 0.49 | 0.42 | 0.33 | 0.56 | 0.72 | 0.55 | 1.00 | 2.84 | 2.38 | 3.54 |
|  | SOCAT+SOCCOM | 0.42 | 0.40 | 0.45 | 0.57 | 0.53 | 0.60 | 0.43 | 0.39 | 0.47 | 0.37 | 0.32 | 0.49 | 0.55 | 0.54 | 0.53 | 2.45 | 2.27 | 2.65 |
|  | SOCCOM-weighted | 0.44 | 0.42 | 0.49 | 0.56 | 0.53 | 0.61 | 0.43 | 0.39 | 0.47 | 0.37 | 0.34 | 0.44 | 0.68 | 0.66 | 0.54 | 2.68 | 2.51 | 2.64 |
|  |  |  |  |  |  |  |  |  |  |  |  |  |  |  |  |  |  |  |  |
| CM2.6 w/o *p*CO_2_ climatology | SOCAT-only | 0.54 | 0.52 | 0.63 | 0.83 | 0.72 | 0.96 | 0.54 | 0.44 | 0.64 | 0.51 | 0.34 | 0.68 | 0.83 | 0.63 | 1.00 | 3.48 | 2.73 | 4.16 |
|  | SOCAT+SOCCOM | 0.47 | 0.46 | 0.50 | 0.72 | 0.65 | 0.79 | 0.48 | 0.41 | 0.52 | 0.44 | 0.32 | 0.62 | 0.61 | 0.59 | 0.48 | 2.88 | 2.51 | 3.04 |
|  | SOCCOM-weighted | 0.51 | 0.47 | 0.58 | 0.73 | 0.68 | 0.81 | 0.49 | 0.42 | 0.56 | 0.43 | 0.34 | 0.60 | 0.67 | 0.73 | 0.50 | 3.01 | 2.82 | 3.10 |
|  |  |  |  |  |  |  |  |  |  |  |  |  |  |  |  |  |  |  |  |
| SOSE | SOCAT-only | 0.29 | 0.27 | 0.34 | 0.37 | 0.39 | 0.39 | 0.22 | 0.21 | 0.22 | 0.12 | 0.10 | 0.14 | 0.99 | 0.71 | 1.14 | 2.75 | 2.09 | 3.13 |
|  | SOCAT+SOCCOM | 0.24 | 0.24 | 0.24 | 0.33 | 0.36 | 0.33 | 0.19 | 0.19 | 0.19 | 0.10 | 0.09 | 0.12 | 1.00 | 0.72 | 1.15 | 2.73 | 2.08 | 3.08 |
|  | SOCCOM-weighted | 0.24 | 0.25 | 0.26 | 0.34 | 0.37 | 0.34 | 0.20 | 0.20 | 0.19 | 0.11 | 0.10 | 0.12 | 0.96 | 0.71 | 1.06 | 2.63 | 2.07 | 2.87 |

**Table S7. Southern Hemisphere (south of 20°S) carbon fluxes from the Jena CarboScope atmospheric inversion^a^**

**2015-2017 Mean fluxes**

| (Pg C yr^-1^) |  | **SOCAT** | **SOCAT + SOCCOM** |
| --- | --- | --- | --- |
| **90°S – 20°S** | Ocean – Contemporary | -1.62 | -1.07 |
|  | Ocean – Anthropogenic | -1.2 | -1.2 |
|  | Ocean – Natural | -0.42 | 0.13 |
|  | Land | 0.11 | -0.43 |

^a^Ocean fluxes are set by the Jena CarboScope mixed layer interpolation scheme. Anthropogenic carbon fluxes are taken from Resplandy et al. (2018) (Figure 5b) and natural carbon fluxes are the difference between the contemporary estimates from this study and the anthropogenic fluxes. The 20°S-90°S region was chosen for comparison with Resplandy et al. (2018). Positive indicates a flux to the atmosphere.
